# Charge Migration in Cations: Structures, Energies, and Vibrational Frequencies of Ground and Excited States of HCCX^+^, CH_3_X^+^, Vinyl Bromide Cation and Propiolic Acid Cations

**DOI:** 10.1021/acs.jpca.6c02409

**Published:** 2026-09-10

**Authors:** Deep Mukherjee, Andrew S. Durden, Matthew R. Hermes, Marco Caricato, H. Bernhard Schlegel

**Affiliations:** † Department of Chemistry, 2954 Wayne State University, Detroit, Michigan 48202, United States; ‡ Department of Chemistry, University of Chicago, Chicago, Illinois 60637, United States; § Department of Chemistry, 4202 University of Kansas, Lawrence, Kansas 66045, United States

## Abstract

Coherent superposition of ground and excited states of cations can produce ultrafast charge migration that can be monitored by attosecond transient absorption, attosecond pump-IR probe, high harmonic spectroscopy, attosecond streaking, and strong field ionization. Vibrational motion on the ground and excited states of the cations leads to decoherence and decay of charge migration. Computational modeling of charge migration requires suitable methods for calculating energy differences between the electronic states of the cations and potential energy surfaces for the ground and excited states of the cations. The performance of CCSD, EOM-CCSD, EOMCC-IP, CASSCF, and MC-PDFT/L-PDFT has been examined for computing ionization energies, cation excitation energies, and harmonic vibrational frequencies for ground- and excited-state cations. Three sets of molecules that are relevant to charge migration were selected to illustrate different aspects of the calculations: HCCI^+^ and HCCBr^+^ have degenerate ground and first excited state of the same symmetry; CH_3_I^+^ and CH_3_Br^+^ are subject to Jahn–Teller distortion in their ground state; charge migration in vinyl bromide cation and propiolic acid cation involves the ground state and second excited state of the cations. CCSD is limited to the lowest energy state of a given symmetry. EOM-CCSD can be used for excited states. Both are easy to apply, “black box” methods. EOMCC-IP is an alternative that is less expensive than EOM-CCSD and is suitable for both ground and excited states of cations with degenerate vibrational modes. CASSCF with state averaging is needed for Jahn–Teller distortion cases. MC-PDFT is suitable for adding correlation energy to CASSCF and generally improves the CASSCF results. L-PDFT is required for the proper treatment of cations with degenerate or closely spaced electronic states.

## Graphical abstract

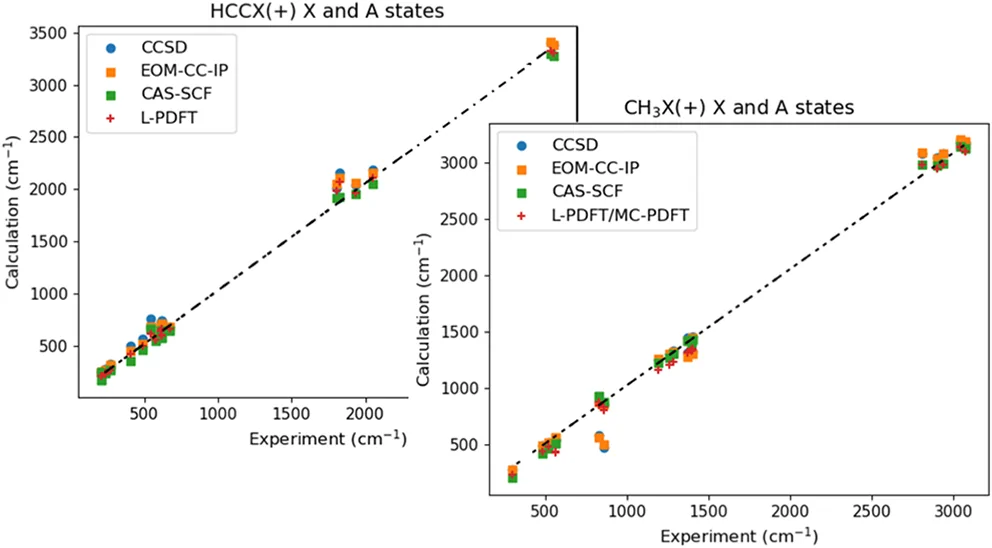

## Introduction

Strong field ionization can produce coherent superpositions of ground and excited state of cations which can result in charge migration.^1–8^ In particular, energy separations of 1–4 eV between electronic states correspond to charge migration with periods of 4 to 1 fs. The electronic dynamics of the superposition is modulated by the dynamics of vibrational wavepackets associated with the states. In the simplest picture, the wavepackets evolve on the potential energy surfaces for the different electronic states. Unless the potential energy surfaces are very similar, the overlap between the evolving wavepackets tends toward zero, leading to decoherence and disappearance of charge migration within a few tens of femtoseconds. Thus, investigations of charge migration in molecular systems and coherence/decoherence of superimposed states require both accurate calculations of the energy difference between the states and the potential energy surfaces of these states to model the vibrational dynamics.

Accurate adiabatic ionization potentials (IPs) can be obtained by coupled-cluster (CC) methods,^9,10^ such as CCSD­(T),^11^ and are applicable to the lowest energy states of each symmetry. Energies of excited states of cations can be calculated by equation of motion coupled cluster (EOMCC).^12,13^ Equation of motion coupled cluster with ionization (EOMCC-IP)^14,15^ yields energies of multiple ionized states with near-coupled-cluster accuracy, but at less cost than EOMCC calculations for each state. For a review of coupled-cluster methods and related approaches, see refs 9,10,12,13 . CC, EOMCC, and EOMCC-IP are “black box” methods that do not need special input from the user. Complete active space self-consistent field (CASSCF)^16^ methods can deal with multiple states but require careful selection of the active space. Dynamic electron correlation can be added to CASSCF calculations by CASPT2 ^17,18^ and NEVPT2,^19^ but this can be costly. Multireference configuration interaction (MRCI)^20^ is an even more costly option for adding dynamic correlation to CASSCF. Multiconfiguration pair-density functional theory (MC-PDFT and L-PDFT)^21–23^ provide lower cost alternatives for adding dynamic correlation to CASSCF methods by using a functional of the on-top density. MC-PDFT incorporates dynamic correlation by augmenting the CASSCF reference energy with an on-top pair-density functional term. L-PDFT builds an effective Hamiltonian in the space of reference states using MC-PDFT-based energies, which makes it especially useful for describing interacting or quasi-degenerate states while retaining the MC-PDFT treatment of correlation. This is particularly important for the degenerate bending modes in HCCX^+^ (X and A states) and CH_3_X^+^ (X state). In this short investigation, we have used CCSD, EOM-CCSD, EOMCCSD-IP, CASSCF, and MC-PDFT/L-PDFT to examine the energies, geometries, and vibrational frequencies of the ground states and lowest excited states of a number of cations relevant to studies of charge migration.

We have selected 6 molecules of interest in charge migration studies to illustrate some of the challenges in calculating potential energy surfaces of cations and their excited states: HCCI^+^, HCCBr^+^, CH_3_I^+^, CH_3_Br^+^, vinyl bromide cation (H_2_C=CHBr^+^), and propiolic acid cation (HC≡CCO_2_H^+^). In haloacetylene cations, the ground state and the first excited state are doubly degenerate; furthermore, the first excited state has the same electronic symmetry as the ground state and cannot be calculated by methods such as CCSD. Methyl halide cations also have degenerate ground states and additionally are subject to Jahn–Teller distortions. In vinyl bromide and propiolic acid, charge migration involves the ground state and the second excited state of the cations. In previous work, we employed CCSD and EOMCC-IP to calculate the energies and vibration frequencies of the X and A states of haloacetylene cations.^24^ In the present work, we use a wider range of methods (CCSD, EOMCC-IP, EOM-CCSD, CASSCF, and MC-PDFT/L-PDFT) to study the ground and excited state potential energy surfaces of a broader range of molecules relevant to charge migration: HCCI, HCCBr, CH_3_I, CH_3_Br, vinyl bromide, and propiolic acid cations (Scheme 1).

**Scheme 1:**
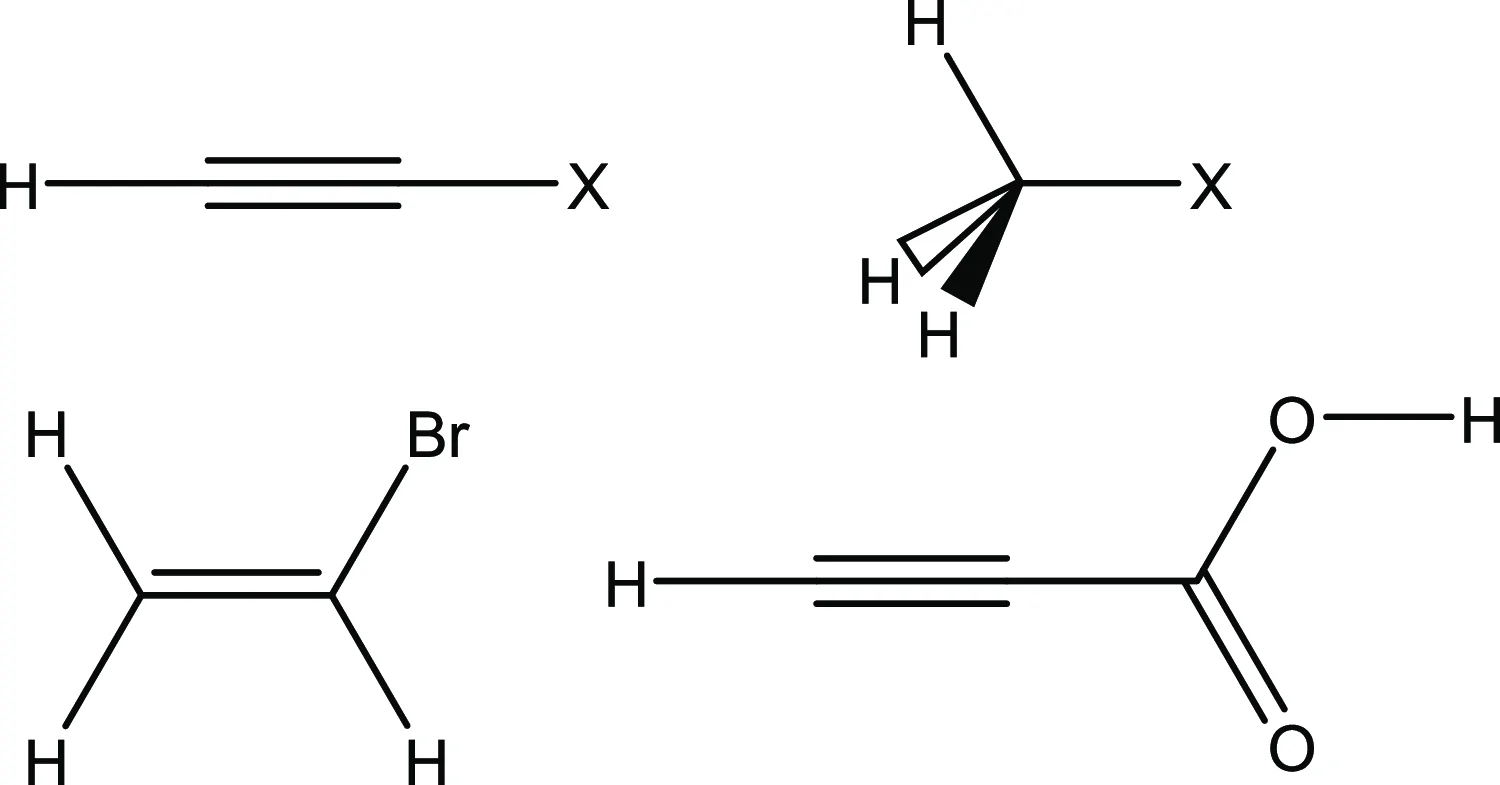
Structures of HCCX (X = I, Br), CH_3_X (X = I, Br), Vinyl Bromide, and Propiolic Acid

## Methods

Calculations with CCSD, CCSD­(T), EOMCC, and EOMCC-IP were carried out with the development version of the Gaussian software package^25^ using the cc-pVXZ basis sets (X = D, T, Q).^26^ The small core cc-pVXZ-pp basis and pseudopotential were used for iodine.^27^ The EOMCC-IP calculations use a wave function with 1h and 2h1p terms. Energies and frequencies with CC methods were calculated with core orbitals frozen (6 for HCCI, 11 for HCCBr, 5 for CH_3_I, 10 for CH_3_Br, 11 for C_2_H_3_Br, and 5 for C_3_O_2_H_2_). For methods without analytic gradients (e.g., EOMCC-IP), geometries were optimized with the Fletcher–Powell approach,^28^ and the second derivatives were calculated by double numerical differentiation of the energies. CASSCF, MC-PDFT, and L-PDFT calculations were carried out with PySCF.^29^ Adiabatic ionization potentials and relative energies of cation states include zero-point vibration corrections.

The CAS-SCF calculations for the selected molecules requires careful selection of the active space. Both the X and A states of the haloacetylene cations are doubly degenerate. Two state- and four state-averaged CASSCF calculations were carried out for the X and A states, respectively. The active space corresponded to 13 electrons in 12 orbitals (CAS­(13,12)). The active space contains three pairs of π and π* orbitals derived from the C≡C π system and the halogen p_π_ orbitals and six σ-type orbitals shown in Scheme 2.

**Scheme 2:**
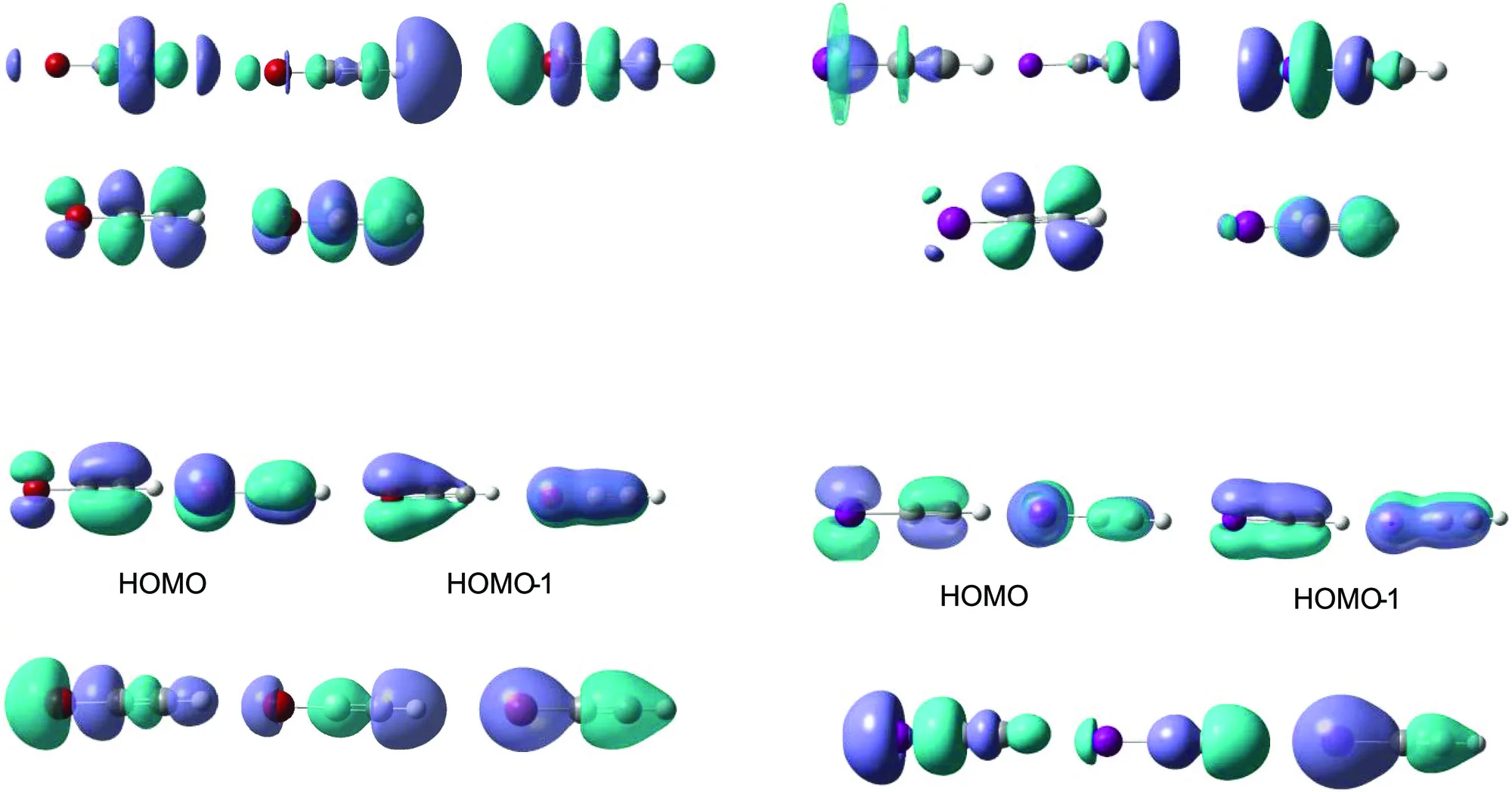
Active Space of CASSCF­(13,12) Calculations for HCCBr^+^ (Left) and HCCI^+^ (Right)

The X states of the methyl halide cations are degenerate, but the A states are not. The X and A states were calculated with two state- and three state-averaged CASSCF, respectively. The active space consisted of 13 electrons in 11 orbitals (CAS­(13,11)) and included two degenerate p-type lone pairs on the halogen, two π-type bonding orbitals on the methyl group, three carbon–halogen sigma bonding orbitals with increasing order of nodes, and four additional antibonding orbitals (Scheme 3).

**Scheme 3:**
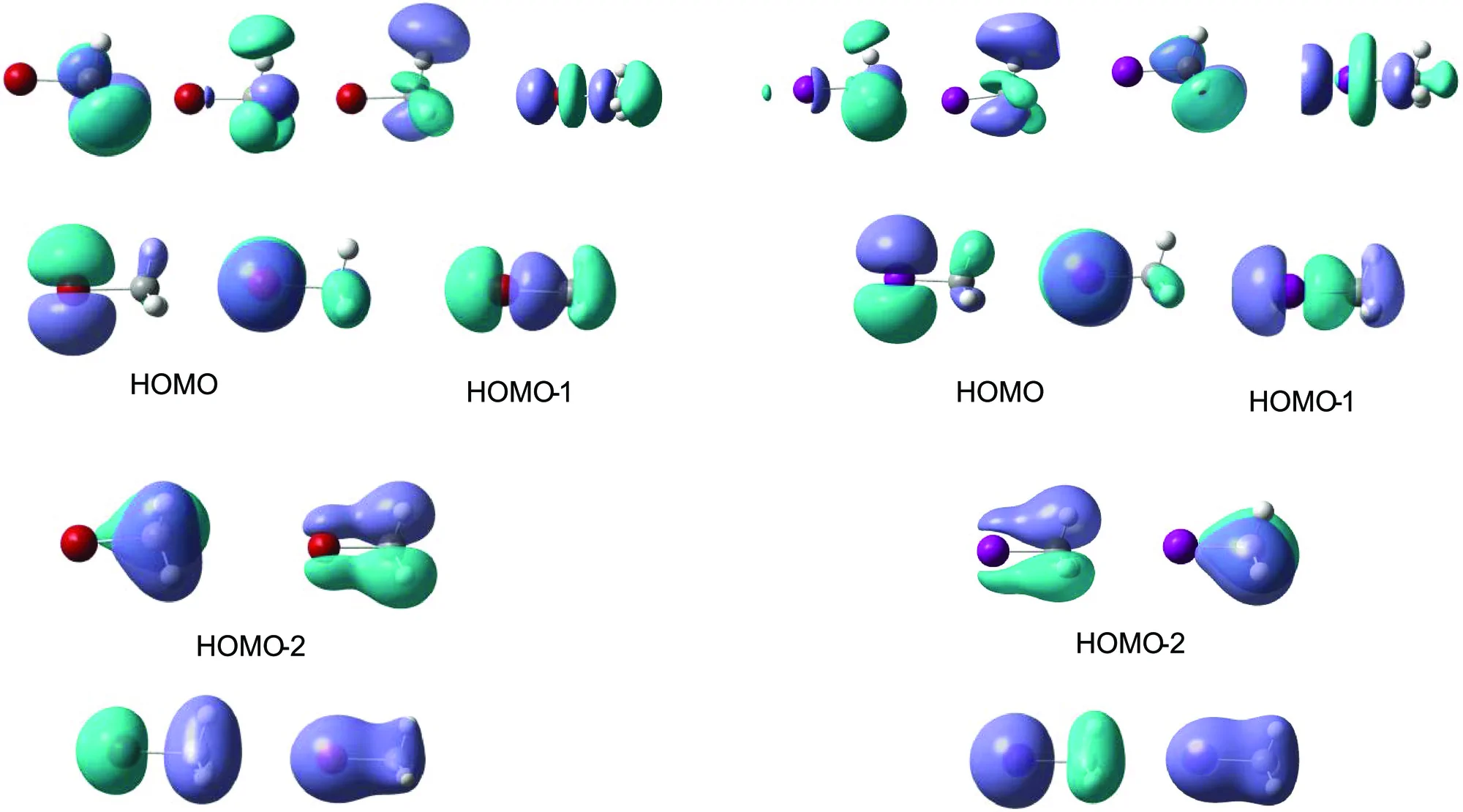
Active Space of CASSCF­(13,11) Calculations for H_3_CBr^+^ (Left) and H_3_CI^+^ (Right)

The X, A, and B states of vinyl bromide cations were computed using CAS­(9,7) averaging over four electronic states. The active space includes two π orbitals formed from linear combinations of the C=C π orbital and the bromine p_π_ orbital and the corresponding π* orbital. The remaining orbitals in the active space are σ orbitals formed from the C–H, C–Br, and C–C σ bonds (Scheme 3). The X, A, and B states of the propiolic acid cations are calculated with CAS­(13,10) averaged over four states. The active space contains the CO π and π* orbitals and the C≡C π and π* orbitals. The other 6 orbitals are the in-plane CC π bond, CC, and CO σ bonds and the lone pair orbitals on both the O atoms (Scheme 4).

**Scheme 4:**
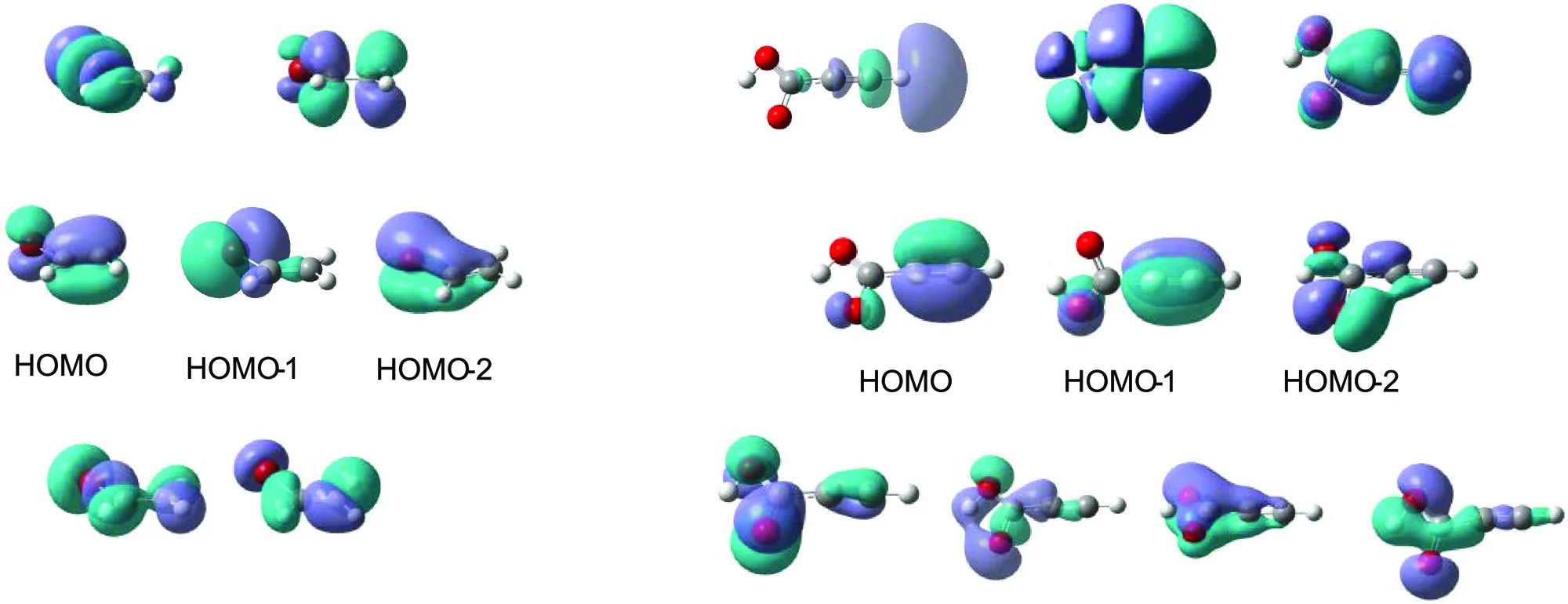
Active Space for the CASSCF­(9,7) Calculation of Vinyl Bromide Cation (Left) and the CASSCF­(13,10) Calculation of Propiolic Acid Cation (Right)

The MC-PDFT/L-PDFT calculations were performed with the on-top PBE functional using the CASSCF calculations discussed above. It is noted that tight thresholds are needed for the CASSCF convergence to obtain accurate wave functions for the MC-PDFT/L-PDFT calculations (1 × 10^–9^ au for the energy and 2 × 10^–6^ au for the gradient). For cases with degenerate electronic states, low-lying excited states or Jahn–Teller effects, MC-PDFT^21,23^ needs to be replaced with L-PDFT to obtain the correct potential energy surface topology.^22,23^ Accurate L-PDFT energies and gradients required large grid sizes (up to (300,1454)) and tight convergence of the Lagrange-multiplier eqs (10^–11^). L-PDFT gradients of degenerate states based on state-averaged CASSCF calculations were taken as the average of the state-specific gradients of the individual states in the degenerate manifold in order to mitigate artifactual symmetry breaking driven by finite precision in seminumerical differentiation of the PDFT energy. The cc-pVDZ basis set^26,27^ was used for the CASSCF, MC-PDFT, and L-PDFT vibrational frequency calculations because numerical differentiation for frequency calculations were too costly with the cc-pVTZ basis set.

To calibrate the methodology, some preliminary frequency calculations were carried out on HCCI and CH_3_I neutral and cations (see Tables S1–S6 in the Supporting Information). These include HF, B3LYP, MP2, CCSD, and EOMCCSD-IP with the cc-pVDZ, cc-pVTZ, and cc-pVQZ basis sets. On average the CCSD frequencies with the cc-pVTZ and cc-pVQZ basis sets differ by about 5 cm^–1^. The cc-pVDZ frequencies are up to 30 cm^–1^ lower than with the cc-pVTZ basis set. EOMCCSD-IP frequencies had to be calculated by numerical differentiation of the energy. Because frequency calculations for propiolic acid require 239 individual CCSD energy calculations for each of the 4 structures, the basis set for propiolic frequencies was limited to cc-VDZ to make the calculations practical.

## Results and Discussion

The experimental and calculated adiabatic ionization potentials (IPs) are compared in Table 1. For HCCX and H_3_CX, the experimental values are the weighted average of the spin states (2/3 ^2^E_3/2_ + 1/3 ^2^E_1/2_); the calculated values are without spin–orbit coupling. The adiabatic CCSD, CCSD­(T), and EOMCC-IP values calculated with the cc-pVTZ basis set have a mean absolute deviation (MAD) of 0.13 eV when compared with the experiment. Calculations with CCSD­(T)/cc-pVQZ reduce the mean absolute deviation to 0.03 eV. The EOMCC-IP results are within 0.1 eV of the CCSD­(T) values with the same basis set. CASSCF IP’s are about 0.5–0.9 eV lower than experiment in part because of the smaller basis set and because of lack of dynamic correlation. Accounting for dynamic electron correlation by MC-PDFT or L-PDFT improves the CASSCF results, yielding values within 0.2–0.6 eV of the experimental results.

**Table 1:** Experimental and Calculated Adiabatic Ionization Energies (eV)

	HCCI	HCCBr	CH_3_I	CH_3_Br	vinyl bromide	propiolic acid
experiment^a^	9.84 ^30^	10.35 ^30^	9.75 ^31^	10.65 ^31^	9.82 ^32^	10.78 ^33,34^
CCSD	9.74	10.19	9.57	10.44	9.63	10.65
CCSD(T)^b^	9.76	10.23	9.61	10.48	9.68	10.67
CCSD(T)^c^	9.85	10.34	9.73	10.61	9.79	10.85
EOMCC-IP	9.81	10.34	9.58	10.46	9.72	10.77^d^
CASSCF^e^	9.06	9.48	9.25	9.76	9.05	9.94
MC-PDFT^e^	9.57	10.10	9.52	10.52	9.59	10.14

a Weighted average of spin–orbit states for HCCX^+^ and CH_3_X^+^.

b CCSD­(T)/cc-pVTZ energy using CCSD/cc-pVTZ-optimized geometry and zero-point energy.

c CCSD­(T)/cc-pVQZ energy using CCSD/cc-pVTZ-optimized geometry and zero-point energy.

d EOMCC-IP/cc-pVTZ energy using EOMCC-IP/cc-pVDZ geometry and zero-point energy.

e cc-pVDZ basis, L-PDFT for HCCX and CH_3_X, MC-PDFT for vinyl bromide and propiolic acid.

The vibrational frequencies for neutral molecules are better established than for cations. Figure 1 compares the calculated frequencies for HCCI, HCCBr, CH_3_I, and CH_3_Br with experiment. The calculated harmonic frequencies are a few percent higher because they do not include anharmonicity corrections. A linear fit with zero intercept (ν_exp_ ≈ scale ν_calc_) yields scale factors of 0.9547, 0.9861, and 0.9857 for CCSD, CASSCF, and state-specific MC-PDFT (*R* ^2^ > 0.999). When the scaled calculated frequencies are compared with experiment, the mean absolute deviations (MAD) are 11, 13, and 37 cm^–1^, and the mean absolute percent deviations (MAPD) are 1.4, 1.8, and 4.2% for CCSD, CASSCF, and MC-PDFT, respectively. The mean absolute deviations for the cations are expected to be somewhat higher because the computational methods may not be as accurate for excited states and because the frequencies for cations are more difficult to measure.

**Figure 1:**
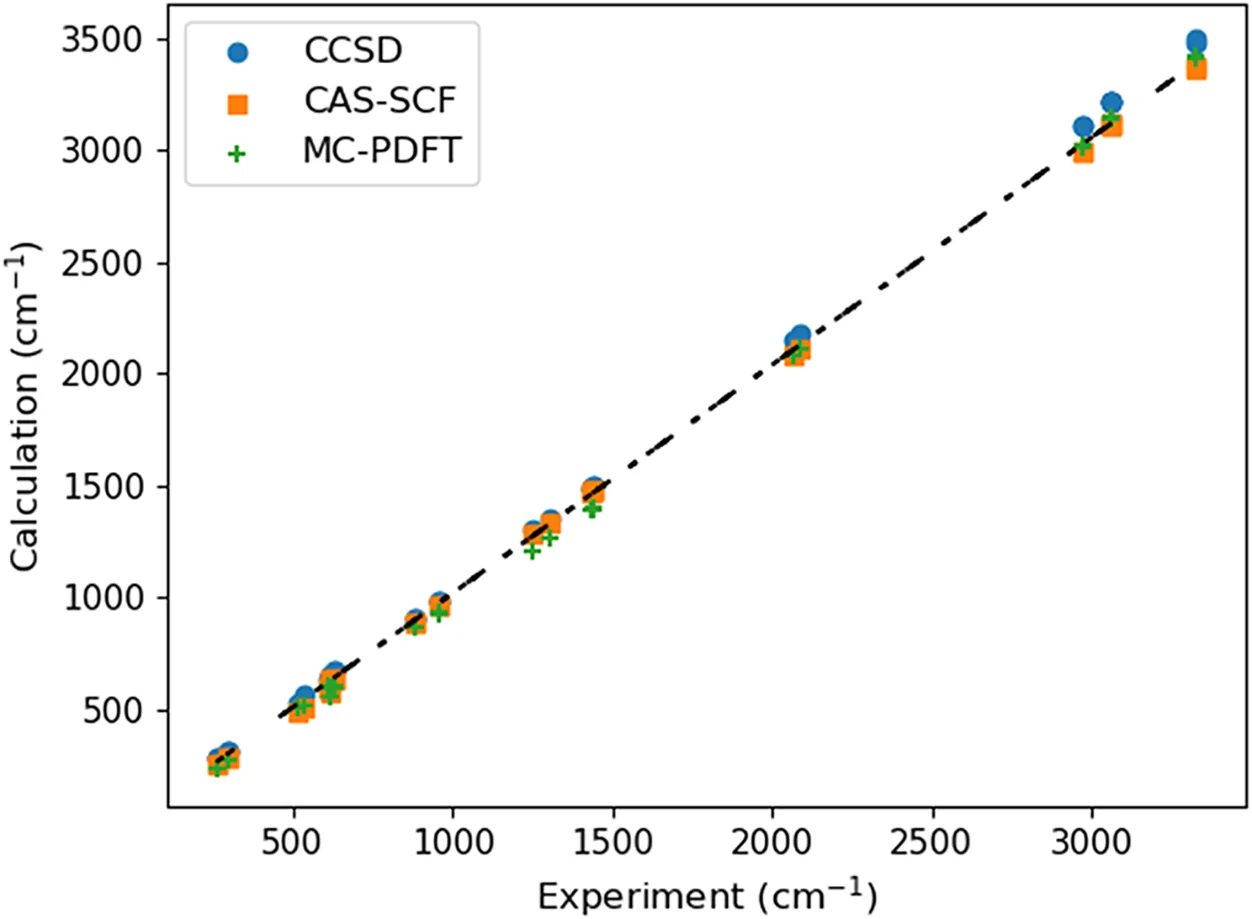
Harmonic vibrational frequencies (cm^–1^) calculated with CCSD, CASSCF, and MC-PDFT compared with experimentally observed frequencies for neutral HCCI, HCCBr, CH_3_I, and CH_3_Br.

The X and A states of HCCX^+^ are generated by removing an electron from the degenerate HOMO and degenerate HOMO–1 (see Scheme 2, antisymmetric and symmetric combination of the C≡C π orbital and the X π-type lone pair orbital, respectively). The coherent superposition of the X and A states of HCCX^+^ results in charge migration between the CC π orbital and the iodine π-type lone pair. The decoherence of the superposition and charge migration in HCCX^+^ have been studied experimentally and computationally;^5,35–37^ the decoherence is due to differences in vibrational motion on the potential energy surfaces of the X and A states. The excitation energies, optimized geometries, and vibrational frequencies for HCCI^+^ and HCCBr^+^ are listed in Table 2. The calculated frequencies are compared with the experimental frequencies in Figure 2. The CCSD and EOMCC-IP data for HCCI^+^ and HCCBr^+^ are from our previous paper.^24^ EOMCC-IP can be used to calculate the optimized geometry and vibrational frequencies for both the X and A states. Because the first excited state (A state) is of the same symmetry as the ground state (X state), the A state cannot be calculated by CCSD and was calculated by EOM-CCSD instead. The adiabatic X to A excitation energies calculated with CC methods (E­(A) – E­(X) in Table 2) are within 0.2 eV of the experimental adiabatic excitation energies. Increasing the basis set from cc-pVTZ to cc-pVQZ changed the EOMCCSD-IP excitation energies by only 0.03 eV for HCCX^+^ and CH_3_X^+^. The CC and CX bond lengths calculated with coupled-cluster methods agree reasonably well with the experimental estimates.^38,39^ The CASSCF bond lengths tend to be a bit longer than the EOMCC-IP values; some shortening is seen when dynamic electron correlation is added by L-PDFT.

**Figure 2:**
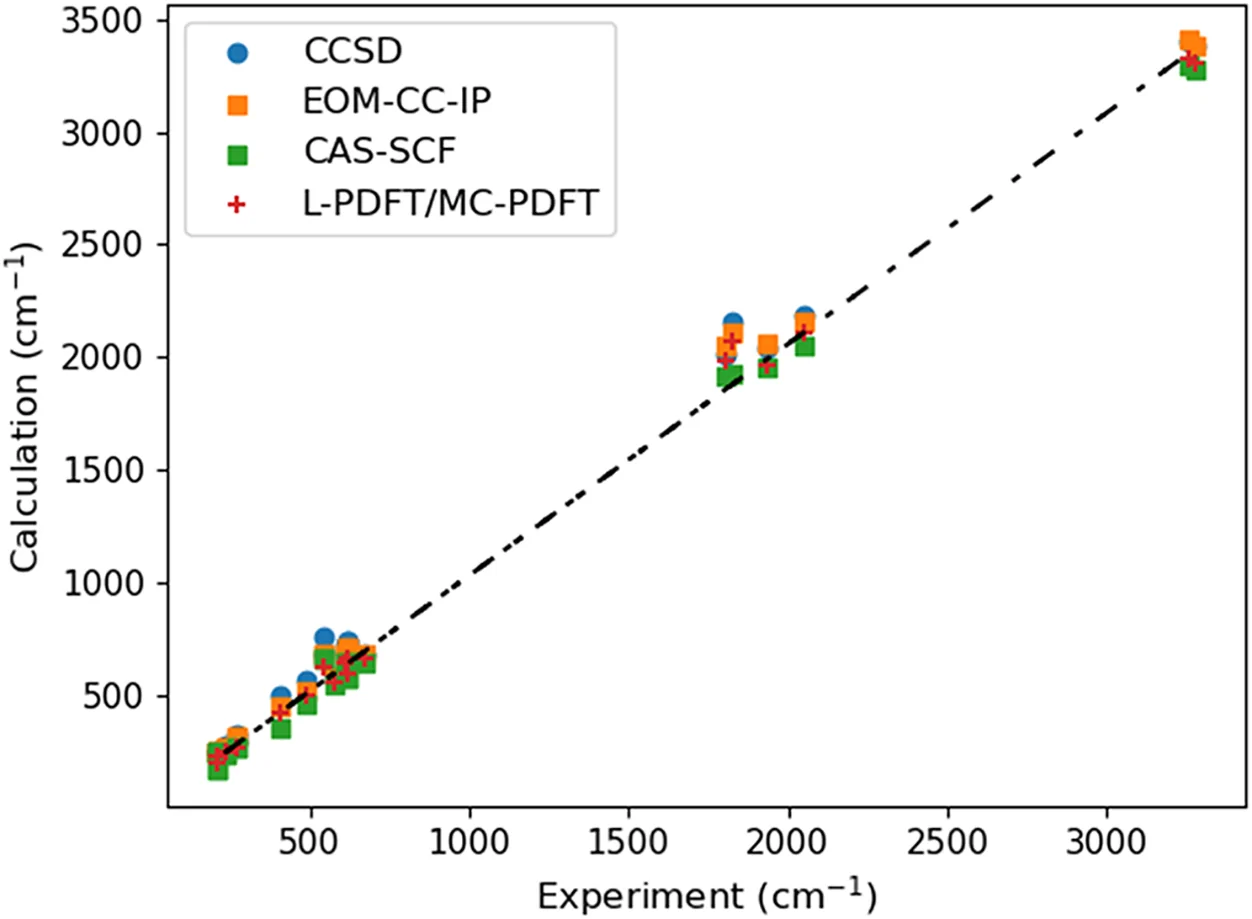
Harmonic vibrational frequencies (cm^–1^) for HCCI and HCCBr cations calculated with CCSD, EOMCC-IP, CASSCF, and L-PDFT compared with experimental frequencies.

**Table 2:** Adiabatic Energy Differences (eV), Bond Lengths (Å), and Harmonic Vibrational Frequencies (cm^–1^) for (a) HCCI and (b) HCCBr Cations

	exp^40^	CCSD(cc-pVTZ)	EOMCC-IP(cc-pVTZ)	CASSCF(cc-pVDZ)	L-PDFT(cc-pVDZ)
(a) HCCl cation					
X state					
R(C–I)	1.919	1.929	1.934	1.966	1.953
R(C≡C)	1.231	1.227	1.222	1.252	1.247
ν_1_ CCI bend	237	256.0	264.8	232.4	247.3
ν_2_ CCI bend	237	270.7	264.8	232.5	247.7
ν_3_ CI str	578	584.5	582.3	541.8	551.3
ν_4_ CCH bend	542	674.7	684.2	663.1	617.1
ν_5_ CCH bend	542	761.2	684.2	663.2	617.7
ν_6_ CC str	1805	2014.6	2046.7	1910.8	1985.5
ν_7_ CH str	3258	3405.3	3406.3	3294.4	3323.4
A state		EOM-CCSD			
E(A) – E(X)	2.15	2.14	2.20(2.23^a^)	2.08	1.87
R(C–I)	2.149	2.100	2.096	2.170	2.125
R(C≡C)	1.217	1.229	1.233	1.259	1.248
ν_1_ CCI bend	212	241.5	249.4	243.7	222.8
ν_2_ CCI bend	212	244.1	249.4	243.9	223.2
ν_3_ CI str	407	493.5	448.9	350.7	423.7
ν_4_ CCH bend	612	691.9	678.3	638.6	637.9
ν_5_ CCH bend	612	718.1	678.3	639.3	638.0
ν_6_ CC str	1822	2154.2	2104.6	1927.6	2064.3
ν_7_ CH str		3409.5	3389.3	3283.5	3323.8
(b) HCCBr cation					
X state					
R(C–Br)		1.723	1.725	1.751	1.741
R(C≡C)		1.238	1.231	1.263	1.253
ν_1_ CCBr bend	273	293.1	311.0	267.5	265.8
ν_2_ CCBr bend	273	320.9	311.0	267.6	266.4
ν_3_ CCH bend	618	669.9	648.4	577.2	594.3
ν_4_ CCH bend	618	741.9	648.4	577.5	595.9
ν_5_ CBr str	673	677.5	680.3	641.7	661.4
ν_6_ CC str	1931	2042.4	2062.1	1953.9	1960.7
ν_7_ CH str	3280	3385.2	3383.2	3275.3	3306.0
A state		EOM-CCSD			
E(A) – E(X)	2.54	2.72	2.78(2.66^a^)	2.61	2.41
R(C–Br)		1.890	1.902	1.962	1.927
R(C≡C)		1.215	1.219	1.245	1.233
ν_1_ CCBr bend	207	220.7	248.8	164.2	199.0
ν_2_ CCBr bend	207	240.8	248.8	164.3	199.1
ν_3_ CBr str	492	563.3	516.9	453.2	494.8
ν_4_ CCH bend	620	714.2	705.3	637.6	663.6
ν_5_ CCH bend	620	737.5	705.3	637.6	663.7
ν_6_ CC str	2051	2180.7	2159.2	2050.6	2106.0
ν_7_ CH str		3421.7	3404.4	3299.6	3336.5

a E­(A) – E­(X) calculated using EOMCC-IP/cc-pVQZ//EOMCC-IP/cc-pVTZ.

Harmonic vibrational frequencies calculated with CCSD, EOMCC-IP, CASSCF, and L-PDFT are about 1–7% higher than the observed (anharmonic) frequencies (see Table 2). The scale factors for the calculated frequencies of the X and A states of HCCI and HCCBr cations for CCSD, EOMCC-IP, CAS-SCF, and L-PDFT are 0.9316, 0.9367, 0.9860, and 0.9674, respectively (*R* ^2^ > 0.997). The MADs for the scaled frequencies with respect to the experiment are 50, 41, 38, and 34 cm^–1^ and the MAPDs are 7.2, 6.4, 7.7, and 4.3%, respectively, for CCSD, EOMCC-IP, CASSCF, and L-PDFT. The EOMCC-IP frequencies are in good agreement with CCSD and EOM-CCSD (MAD = 23 cm^–1^). For the A state of HCCBr^+^, some of the frequencies calculated with CASSCF are too low because the C–Br bond is too long. With the L-PDFT approach, the C–Br bond length and the vibrational frequencies are in better agreement with EOMCC-IP. Since the HCCX cations are linear, they should have degenerate bending vibrations. However, spin-unrestricted, single-reference CCSD and EOM-CCSD calculations for these cations do not have degenerate bending modes. This is because bends parallel and perpendicular to the orbital with the unpaired electron see different potentials. Complex unrestricted CCSD would be an alternative, but very few codes have this capability. EOMCC-IP calculations do yield degenerate frequencies for the bending modes because the calculations are based on the orbitals of the neutral. Degenerate frequencies are also obtained with state-averaged CASSCF calculations for the cations. MC-PDFT calculations with standard integration grids based on state-averaged CASSCF results can have splittings of the degenerate frequencies as large as 120 cm^–1^. When L-PDFT calculations are used with very large integration grids, the splittings are 1 cm^–1^ or less for the degenerate bends.

Similar to the haloacetylenes, the potential energy surfaces for ground- and excited-state methyl halides cations are needed for the interpretation of studies dealing with strong field ionization, charge migration, and molecular dynamics. The X and A states of CH_3_X^+^ are generated by removing an electron from the degenerate HOMO and nondegenerate HOMO–1 (see Scheme 3, π-type and σ-type lone pair orbitals on the halogen, respectively). The excitation energies, optimized geometries and vibrational frequencies for CH_3_I^+^ and CH_3_Br^+^ are listed in Table 3. The calculated frequencies are compared with the experimental frequencies in Figure 3. The mean absolute percent deviations of the scaled frequencies compared to experiment are 5.5, 5.4, 4.7, and 6.0%, respectively. In *C*_3*v*_ symmetry, these cations have degenerate electronic ground states and are subject to the Jahn–Teller effect. This involves vibronic interactions between the *E* symmetry electronic wave functions and the *e* symmetry vibrational modes. Jahn–Teller effects in methyl halide cations states have been discussed in the literature^41^ and a detailed treatment beyond the scope of the present study. For single-reference calculations, geometry optimization of the X state of CH_3_X^+^ breaks *C*_3*v*_ symmetry and leads to a *C*_*s*_ symmetry minimum with *A*′ electronic symmetry. For the CCSD, EOM-CCSD, and EOMCC-IP calculations of the *A*′ symmetry X state, the largest effect is for the *a*″ rocking mode with smaller effects for the *a*″ deformation and *a*″ asymmetric CH stretch. The *a*′ vibrational modes are in good agreement with the experimental values. The CASSCF calculations are for a state average of the two X states and lead to a *C*_3*v*_ symmetry geometry but with somewhat longer C-X bonds. The L-PDFT calculations, built on the state-averaged CASSCF wave function, maintains *C*_3*v*_ symmetry, improves the C-X bond length, and has splittings in the degenerate vibrational frequencies of less than 0.5 cm^–1^.

**Figure 3:**
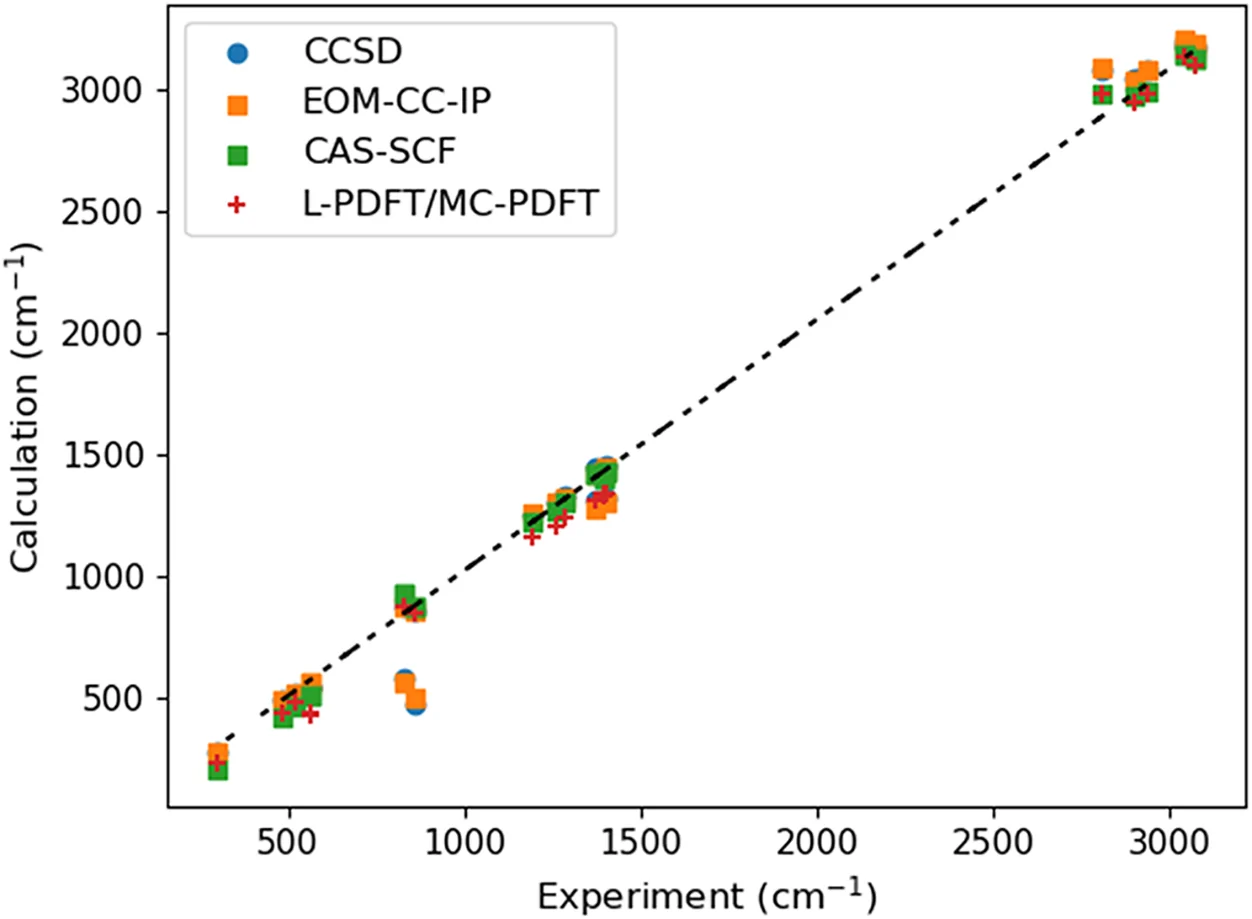
Harmonic vibrational frequencies (cm^–1^) for CH_3_I and CH_3_Br cations calculated with CCSD, EOMCC-IP, CASSCF, and L-PDFT/MC-PDFT compared with experimental frequencies.

**Table 3:** Adiabatic Energy Differences (eV), Bond Lengths (Å), and Harmonic Vibrational Frequencies (cm^–1^) for (a) CH_3_I and (b) CH_3_Br Cations

	exp^42,44–49^	CCSD(cc-pVTZ)	EOMCC-IP(cc-pVTZ)	CASSCF(cc-pVDZ)	L-PDFT(cc-pVDZ)
(a) CH_3_I cation					
X state (C_s_)					
R(C–I)	2.126	2.137	2.140	2.217	2.180
<H_1_CI	108.3	103.7	103.4	104.8	104.9
<H_2_CI, <H_3_CI	108.3	106.7	106.6	104.8	104.9
ν_1_ *a*′ CI str	480	498.2	491.4	427.6	439.6
ν_2_ *a*″ CH_3_ rock	861	481.6	505.0	882.6	847.6
ν_3_ *a*′ CH_3_ rock	861	860.3	862.1	882.6	847.8
ν_4_ *a*′ CH_3_ umbrella	1257	1306.0	1302.7	1269.3	1212.6
ν_5_ *a*″ CH_3_ deform	1399	1325.6	1304.4	1428.7	1342.2
ν_6_ *a*′ CH_3_ deform	1399	1453.6	1450.4	1428.8	1342.5
ν_7_ *a*′ CH_3_ sym-str	2938	3078.2	3077.1	2987.0	2984.4
ν_8_ *a*″ CH_3_ asym-str	3043	3181.0	3184.4	3138.5	3136.5
ν_9_ *a*′ CH_3_ asym-str	3043	3197.0	3200.4	3138.5	3136.7
A state (*C*_3*v*_)					MC-PDFT
E(A) – E(X)	2.09	2.03	2.15(2.12^a^)	1.96	1.97
R(C–I)	2.503	2.511	2.496	2.679	2.625
<HCI	98.2	95.0	95.1	93.9	94.2
ν_1_ CI str	294	283.5	284.5	206.8	235.3
ν_2_ CH_3_ rock	565^b^	550.6	569.9	512.1	436.3
ν_3_ CH_3_ rock	565^b^	550.6	570.0	512.1	438.6
ν_4_ CH_3_ umbrella	1192	1249.5	1266.1	1230.1	1168.1
ν_5_ CH_3_ deform	1394	1432.8	1434.6	1401.0	1334.3
ν_6_ CH_3_ deform	1394	1432.8	1434.8	1401.0	1339.6
ν_7_ CH_3_ sym-str	2810	3080.7	3084.5	2983.0	2980.9
ν_8_ CH_3_ asym-str		3268.3	3276.2	3189.1	3184.7
ν_9_ CH_3_ asym-str		3268.3	3276.3	3189.2	3187.6
					

	exp^31^	CCSD(cc-pVTZ)	EOMCC-IP(cc-pVTZ)	CASSCF(cc-pVDZ)	L-PDFT(cc-pVDZ)
(b) CH_3_Br cation					
X state (C_s_)					
R(C–Br)		1.942	1.943	2.009	1.957
<H_1_CBr		102.6	102.5	104.5	105.2
<H_2_CBr, <H_3_CBr		106.8	107.0	104.5	105.2
ν_1_ CBr str	518	524.5	522.4	464.6	486.8
ν_2_ CH_3_ rock	828	587.5	567.9	931.9	876.9
ν_3_ CH_3_ rock	828	882.1	880.4	931.9	877.9
ν_4_ CH_3_ umbrella	1282	1329.4	1322.2	1306.2	1240.2
ν_5_ CH_3_ deform	1368	1311.4	1279.4	1418.3	1317.8
ν_6_ CH_3_ deform	1368	1446.7	1442.7	1418.4	1318.3
ν_7_ CH_3_ sym-str	2899	3042.9	3031.3	2973.9	2948.8
ν_8_ CH_3_ asym-str	3077	3171.9	3163.0	3127.9	3097.0
ν_9_ CH_3_ asym-str	3077	3174.7	3183.4	3128.0	3097.6
A state(*C*_3*v*_)					MC-PDFT
E(A) – E(X)	2.45	1.97	2.09(2.09^a^)	1.71	2.01
R(C–Br)		2.414	2.392	3.222	2.427
<HCBr		93.6	93.7	90.3	93.7
ν_1_ CBr str		240.2	255.1	45.2	245.4
ν_2_ CH_3_ rock		527.0	537.7	156.9	439.3
ν_3_ CH_3_ rock		527.0	537.8	164.9	440.7
ν_4_ CH_3_ umbrella		1313.6	1326.1	1258.2	1224.3
ν_5_ CH_3_ deform		1435.7	1436.4	1269.6	1338.2
ν_6_ CH_3_ deform		1435.7	1437.4	1360.3	1341.4
ν_7_ CH_3_ sym-str		3076.0	3082.2	2976.3	2969.7
ν_8_ CH_3_ asym-str		3268.2	3279.1	3118.7	3174.7
ν_9_ CH_3_ asym-str		3268.2	3279.4	3127.9	3175.4

a E­(A) – E­(X) calculated using EOMCC-IP/cc-pVQZ//EOMCC-IP/cc-pVTZ.

b Reported experimental value 1130 cm^–1^ appears to be an overtone, see text for discussion.

The analysis of the A states of the methyl halide cations is simpler because they are not degenerate and not affected by Jahn–Teller interactions. The CCSD/cc-pVTZ calculations should yield reliable frequencies. The EOMCC-IP frequencies are in good agreement with the CCSD values (MAD = 10 cm^–1^), further validating the use of EOMCC-IP for the vibrational frequencies of excited cations. The experimentally determined C–I bond length in the A state of CH_3_I^+^ (2.503 Å) is considerably longer than for the X state (2.126 Å) and the CH_3_ group is nearly flat (∠HCI = 98.2°).^42^ The C–I bond lengths calculated by CCSD and EOMCC-IP (2.511 and 2.496 Å, respectively), are in good agreement with experiment. The C–I bond lengths calculated by CASSCF (2.679 Å with CAS­(13,11), 2.770 Å with CAS­(11,10), and 2.778 Å with CAS­(9,8)) and L-PDFT (2.625 Å) are significantly longer. Banares and co-workers^43^ obtained R­(C–I) = 2.599 Å using CAS­(11,10) with spin–orbit coupling and an all-electron basis, suggesting that relativistic effects may also be important for the geometry. The longer C–I bond in the A state leads to a significant decrease in the C–I stretching frequency for the A state compared to the X state (294 cm^–1^ exp for A vs 480 cm^–1^ exp for X). The experimental frequency for rocking mode in the A state,^44^ 1130 cm^–1^, was tentatively assigned based on a progression in the MATI-PD spectrum.^45^ The corresponding frequencies calculated by CCSD, EOMCC-IP, CASSCF, and MC-PDFT (551, 570, 512, 437 cm^–1^, respectively) agree fairly well with each other but are a factor of 2 smaller. This suggests 1130 cm^–1^ is the first overtone of the rocking mode. Hence, we have tentatively listed 1130/2 = 565 cm^–1^ in Table 3a as the experimental value for the rocking mode in the A state of CH_3_I^+^. The calculated harmonic C–H stretching modes for HCCX^+^ and the X states of CH_3_X^+^ are approximately 4–5% higher than the observed anharmonic frequencies. By contrast, the symmetric C–H stretch frequencies for the A state of CH_3_I^+^ calculated with CCSD (3081 cm^–1^) is 10% higher than the reported experimental value (2810 cm^–1^). This may be a misassignment or a strong resonant interaction with an overtone; the most likely candidate is the overtone of the deformation mode, 2 × 1394 cm^–1^.

No experimental data are available for comparison with the calculated frequencies for the A state of CH_3_Br. Since the CCSD and EOMCC-IP results for the A state of CH_3_I^+^ are in good agreement with experiment, the CASSCF and MC-PDFT calculations for CH_3_Br^+^ are compared with the corresponding CCSD and EOMCC-IP values. The C–Br bond length calculated by CASSCF is much longer than with CCSD and EOMCC-IP (3.222 Å vs 2.414 Å, 2.392 Å) and the CH_3_ group is flatter (90.3° vs 93.6°, 93.7°). As a result, the C–Br stretching, rocking, and deformation frequencies are as much as 370 cm^–1^ lower than the CCSD frequencies. The MC-PDFT calculations greatly improve the geometry and bring the vibrational frequencies in line with the CCSD results (MAPD = 7.2% for MC-PDFT vs 28.1% for CASSCF).

The experimental vibrational frequencies of neutral vinyl bromide^50^ and propiolic acid^51^ are compared with the CCSD, CAS-SCF, and MC-PDFT calculations in Figure 4. The scale factors for the frequencies calculated with CCSD, CASSCF, and MC-PDFT are 0.9531, 0.9430, and 0.9828, respectively (*R* ^2^ > 0.999). When the scaled calculated frequencies are compared with experiment, the mean absolute deviations are 8, 40, and 32 cm^–1^, and the mean absolute percent deviations are 0.9, 3.5, and 2.9% for CCSD, CASSCF, and MC-PDFT, respectively.

**Figure 4:**
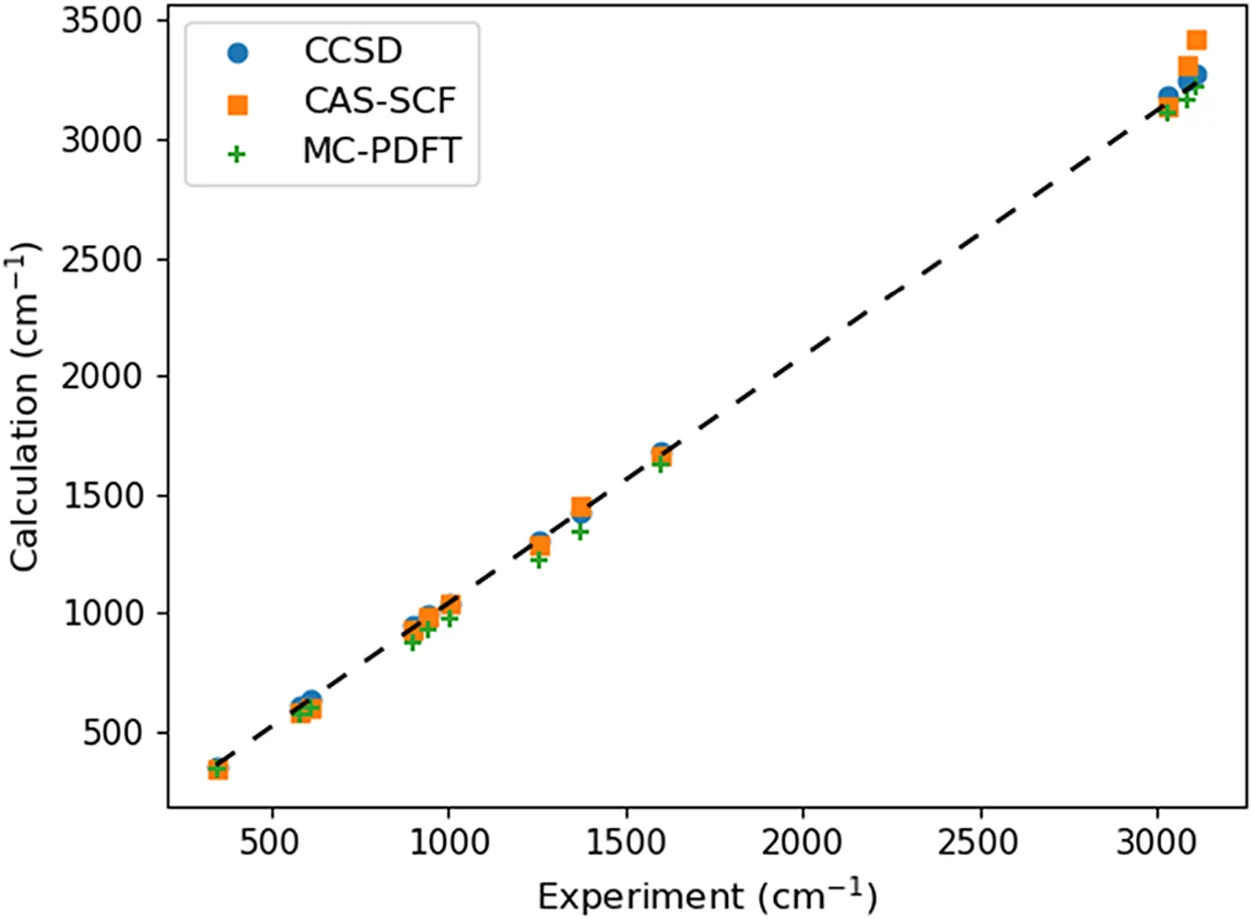
Harmonic vibrational frequencies (cm^–1^) for neutral vinyl bromide and propiolic acid calculated with CCSD, CASSCF, and MC-PDFT compared with experimentally observed frequencies.

The excitation energies, optimized geometries and vibrational frequencies for vinyl bromide cations (CH_2_=CHBr^+^) are listed in Table 4. The X, A, and B states are generated by removing an electron from HOMO, HOMO–1, and HOMO–2 (see Scheme 4, the antisymmetric combination of the C=C π orbital and the out-of-plane Br π-type lone pair orbital, the in-plane Br π-type lone pair orbital, and the symmetric combination of the C=C π orbital and the out-of-plane Br π-type lone pair orbital, respectively). Since these cations do not have degenerate electronic states, the analysis is somewhat simpler. However, both the first and second excited states of these cations are needed because charge migration in both molecules involves the B state which is the same symmetry as the X state.

**Table 4:** Adiabatic Energy Differences (eV), Bond Lengths (Å), and Harmonic Vibrational Frequencies (cm^–1^) for Vinyl Bromide Cations

	exp^a^	CCSD(cc-pVTZ)	EOMCCSD(cc-pVTZ)	EOMCC-IP(cc-pVTZ)	CASSCF(cc-pVDZ)	MC-PDFT(cc-pVDZ)
X state						
R(C=C)		1.386		1.383	1.384	1.385
R(C–Br)		1.794		1.837	1.906	1.832
<CCBr		121.1		120.3	122.1	122.4
ν_1_ *a*′ CCBr bend	348	351.4		355.5	360.8	343.7
ν_2_ *a*″ CBr out-of-plane bend	392	418.6		436.0	442.0	404.3
ν_3_ *a*′ CBr str	700	734.9		719.4	669.7	679.1
ν_4_ *a*″ CH out-of-plane bend	838	885.0		896.1	931.2	840.7
ν_5_ *a*″ CH_2_ out-of-plane bend	1037	1045.6		1068.2	1059.0	996.1
ν_6_ *a*′ CH_2_ in-plane bend	1037	1060.4		1060.7	1095.6	1033.4
ν_7_ *a*′ CH in-plane bend	1258	1300.0		1312.9	1358.4	1248.3
ν_8_ *a*′ CH_2_ in-plane bend	1309	1378.4		1386.7	1425.2	1305.3
ν_9_ *a*′ CC str	1470	1535.7		1557.6	1614.2	1458.9
ν_10_ *a*′ CH str		3168.0		3174.3	3318.7	3100.9
ν_11_ *a*′ CH str		3231.0		3239.5	3381.8	3170.6
ν_12_ *a*′ CH str		3284.7		3291.2	3465.4	3215.8
A state						
E(A) – E(X)	1.10	1.06	1.18	1.04(1.08^b^)	1.22	0.84
R(C=C)		1.318	1.307	1.321	1.328	1.327
R(C–Br)		1.939	1.982	1.938	2.052	2.023
<CCBr		119.9	118.0	119.4	124.7	124.6
ν_1_ *a*′ CCBr bend	270	284.4	272.5	271.4	284.0	258.1
ν_2_ *a*′ CBr str	468	483.3	480.2	480.5	435.0	396.3
ν_3_ *a*″ CBr out-of-plane bend	566	636.5	588.0	578.6	547.3	550.2
ν_4_ *a*″ CH out-of-plane bend	844	919.3	933.1	893.5	852.3	823.4
ν_5_ *a*′ CH_2_ in-plane bend		960.4	948.7	952.8	981.5	872.3
ν_6_ *a*″ CH_2_ out-of-plane bend	1002	1029.7	1031.4	1015.4	986.2	919.5
ν_7_ *a*′ CH in-plane bend	1193	1237.1	1246.6	1230.7	1285.3	1162.5
ν_8_ *a*′ CH_2_ in-plane bend		1435.6	1428.5	1422.1	1463.8	1330.5
ν_9_ *a*′ CC str	1566	1746.0	1736.3	1651.2	1675.1	1592.8
ν_10_ *a*′ CH str		3174.6	3170.0	3176.0	3321.2	3093.4
ν_11_ *a*′ CH str		3229.4	3249.4	3225.2	3402.2	3137.4
ν_12_ *a*′ CH str		3279.0	3275.2	3284.2	3437.8	3213.4
B state						
E(B) – E(X)	2.26		2.40	2.35(2.37^b^)	2.22	2.05
R(C=C)			1.341	1.335	1.369	1.355
R(C–Br)			2.050	1.925	2.156	2.176
<CCBr			120.3	120.8	124.6	124.6
<HCCBr			9.8	14.4	22.4	22.4
ν_1_ *a*′ CCBr bend	292		307.6	305.0	256.5	296.1
ν_2_ *a*′ CBr str	413		459.3	465.4	291.0	436.4
ν_3_ *a*″ CBr out-of-plane bend	557		523.0	594.5	480.0	536.9
ν_4_ *a*″ CH out-of-plane bend	644		644.5	799.5	650.4	660.7
ν_5_ *a*′ CH_2_ in-plane bend	854		935.9	885.1	972.5	872.5
ν_6_ *a*″ CH_2_ out-of-plane bend	1014		1010.3	982.6	1012.8	943.5
ν_7_ *a*′ CH in-plane bend	994		1218.2	1145.5	1281.2	1176.1
ν_8_ *a*′ CH_2_ in-plane bend	1306		1400.4	1366.5	1457.3	1276.3
ν_9_ *a*′ CC str	1427		1643.3	1545.4	1629.2	1673.0
ν_10_ *a*′ CH str			3117.7	3077.8	3287.4	3003.8
ν_11_ *a*′ CH str			3232.7	3193.9	3381.4	3140.4
ν_12_ *a*′ CH str			3240.0	3202.0	3431.6	3153.9

a Experimental adiabatic excitation energies and vibrational frequencies from refs 32,52,53 .

b E­(A) – E­(X) calculated using EOMCC-IP/cc-pVQZ//EOMCC-IP/cc-pVTZ.

The experimental adiabatic ionization energies and vibrational frequencies of the X, A, and B states of vinyl bromide cation have been carefully investigated by Lee and Kim.^32,52,53^ The excitation energies calculated with CCSD, EOMCCSD, and EOMCC-IP agree with the experimental values to ±0.15 eV. Increasing the basis set from cc-pVTZ to cc-pVQZ changed the EOMCCSD-IP excitation energies by 0.02–0.04 eV. Excitation from the X state to the A and B states results in shorter C=C bonds and longer C–Br bonds. CASSCF yields somewhat longer bonds than CC methods; adding dynamic correlation with MC-PDFT brings the bond lengths closer to the values obtained with CC methods. As noted by Lee and Kim^53^ from their TD-DFT calculations, the B state is twisted about the C=C bond. In the present CC, CASSCF, and MC-PDFT calculations, the B state is twisted by 10–22° and is about 0.1 eV lower in energy than the planar geometry.

The excitation from the X state to the B state (2.26 eV exp, 2.05–2.40 eV calc) is comparable to the energy for dissociation to C_2_H_3_^+^ + Br (2.10 eV exp, 2.1–2.25 eV calc. relative to the X state). Experimentally, the B state has discrete peaks in the vibrational spectrum indicating that it is bound.^53^ MRCI calculations by Yamaguchi^54^ showed that the B state can dissociate directly over a small barrier of 0.22–0.34 eV. Direct dissociation has also been observed for vibrationally excited A state.^55^ Real-time integration of TD-DFT and Ehrenfest dynamics have been used to simulate strong field ionization to produce a coherent superposition of cation states and charge migration in vinyl bromide.^56^

Experimental frequencies are available for only some of the vibrational modes of the X, A, and B states of vinyl bromide cations. EOMCC-IP provides a uniform level of theory for all of the vibrational frequencies. Figure 5 compares the experimental and calculated frequencies with those computed by EOMCC-IP. Using the available experimental data, the scale factors for frequencies calculated with CCSD/EOM-CCSD, EOMCC-IP, CAS-SCF, and MC-PDFT are 0.9421, 0.9474, 0.9057, and 0.9740, respectively (*R* ^2^ > 0.998). The MAPDs of the scaled frequencies compared to experiment are 3.8, 3.5, 8.7, and 5.5%, respectively.

**Figure 5:**
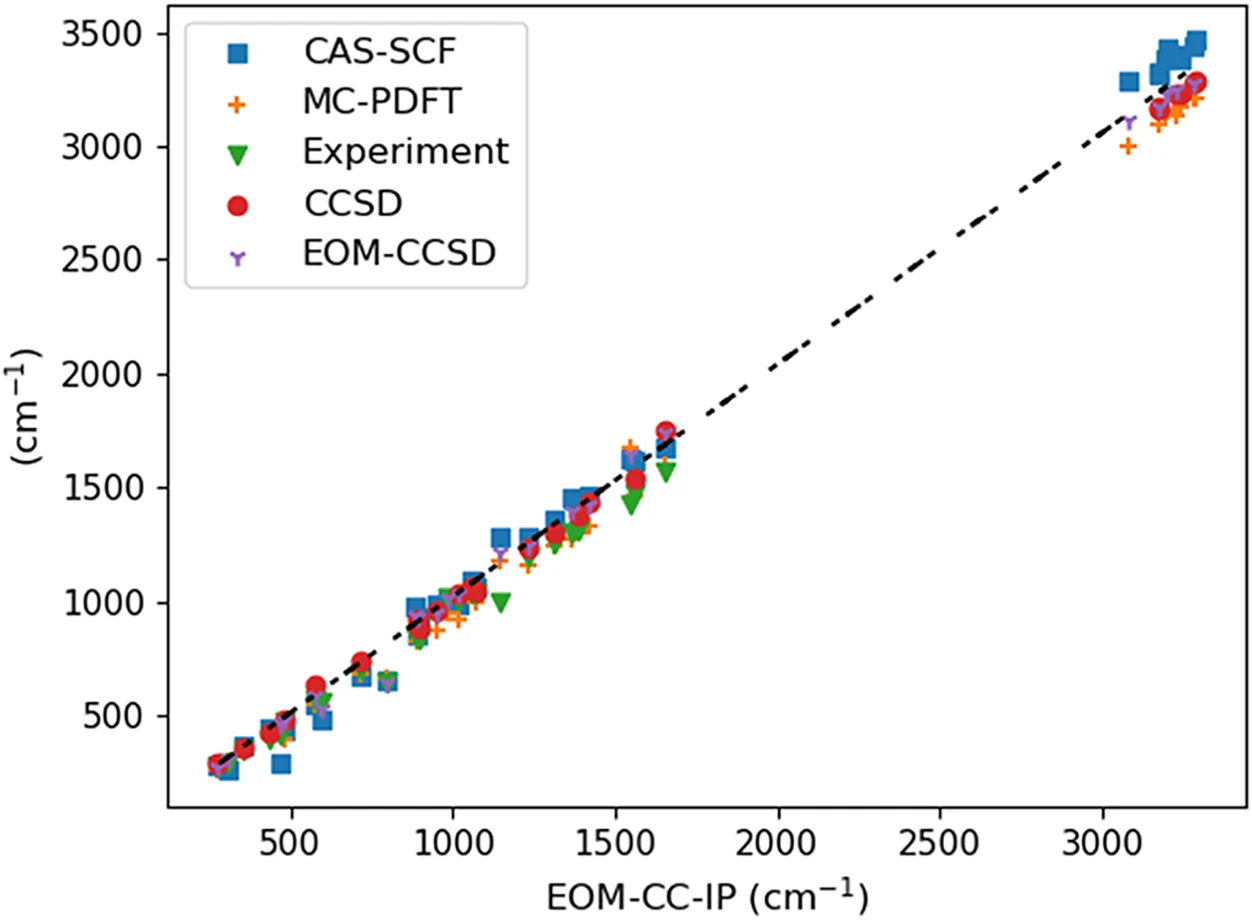
Harmonic vibrational frequencies (cm^–1^) for vinyl bromide cations calculated with CCSD, CASSCF, MC-PDFT, and EOM-CCSD and experimentally observed frequencies, compared with calculated EOMCC-IP frequencies for X, A, and B states of the vinyl bromide cation.

Charge migration in propiolic acid has been investigated by a number of methods.^57–60^ A full quantum dynamical study^57^ on a potential calculated at the ADC(3)/DZP level of theory has shown that the coherent superposition of the X and B states of propiolic acid cation has a coherence lifetime of 15–20 fs. The excitation energies, optimized geometries, and vibrational frequencies for propiolic acid cations are listed in Table 5. The electronic symmetry of the X state is *A*′ and for the A state is *A*″. These states are formed by ionization from the in-plane and out-of-plane π orbitals of the CC triple bond, respectively (HOMO and HOMO–1, see Scheme 4). The *A*′ symmetry B state results from ionization of the in-plane carbonyl oxygen orbital (HOMO–2, Scheme 4).

**Table 5:** Adiabatic Energy Differences (eV), Bond Lengths (Å), and Harmonic Vibrational Frequencies (cm^–1^) for Propiolic Acid Cations^a^

	CCSD(cc-pVTZ)	EOMCCSD(cc-pVTZ)	EOMCC-IP(cc-pVDZ)	CASSCF(cc-pVDZ)	L-PDFT(cc-pVDZ)
X state					
E_rel_	0.00		0.00	0.00	0.00
R(C≡C)	1.210		1.234	1.222	1.223
R(C–C)	1.407		1.429	1.415	1.429
R(C–O)	1.276		1.282	1.262	1.300
R(C=O)	1.266		1.251	1.245	1.234
ν_1_ *a*′ in-plane CCC bend	133.5		143.0	204.2	138.6
ν_2_ *a*″ out-of-plane CCC bend	210.2		211.2	273.2	223.3
ν_3_ *a*′ CC=O bend	401.5		427.4	508.2	416.8
ν_4_ *a*′ CC–O bend	530.9		513.6	545.0	519.9
ν_5_ *a*″ OH torsion	619.6		608.5	626.4	609.2
ν_6_ *a*″ CCO2 out-of-plane	745.1		735.3	772.8	734.5
ν_7_ *a*′ in-plane CH bend	768.0		755.3	840.2	760.1
ν_8_ *a*″ out-of-plane CH bend	798.4		761.9	841.5	767.1
ν_9_ *a*′ C–C str	889.7		879.0	923.1	861.5
ν_10_ *a*′ OH bend	1199.7		1227.9	1302.6	1111.8
ν_11_ *a*′ C–O str	1412.7		1403.9	1437.9	1327.4
ν_12_ *a*′ C=O str	1598.1		1656.3	2051.6	1477.8
ν_13_ *a*′ C≡C str	2265.1		2166.9	2263.2	2192.3
ν_14_ *a*′ CH str	3422.3		3395.6	3527.6	3364.7
ν_15_ *a*′ OH str	3700.0		3686.5	3973.9	3551.0
A state					
E(A) – E(X)	0.59	0.66	0.82(0.74^b^)	0.83	0.73
R(C≡C)	1.249	1.249	1.253	1.235	1.223
R(C–C)	1.497	1.512	1.464	1.444	1.421
R(C–O)	1.309	1.312	1.305	1.312	1.296
R(C=O)	1.193	1.187	1.230	1.228	1.292
ν_1_ *a*′ in-plane CCC bend	168.8	170.1	163.1	200.2	167.6
ν_2_ *a*″ out-of-plane CCC bend	257.7	259.3	257.1	295.4	227.5
ν_3_ *a*′ CC–O bend	512.3	508.8	398.4	538.3	459.4
ν_4_ *a*′ CC=O bend	537.6	552.8	554.3	613.1	552.3
ν_5_ *a*″ OH torsion	596.2	597.4	616.7	640.0	692.4
ν_6_ *a*′ C–C str	744.9	748.8	602.4	757.2	712.0
ν_7_ *a*′ in-plane CH bend	646.7	670.3	645.0	762.6	753.8
ν_8_ *a*″ CCO2 out-of-plane	737.8	736.5	732.9	832.0	810.8
ν_9_ *a*″ out-of-plane CH bend	789.5	797.0	816.4	906.4	848.8
ν_10_ *a*′ C–O str	1201.6	1200.1	1111.1	1101.5	1105.9
ν_11_ *a*′ OH bend	1365.1	1372.3	1261.5	1401.8	1249.2
ν_12_ *a*′ C=O str – C–O str	1782.3	1866.1	1575.9	1630.8	1414.1
ν_13_ *a*′ C≡C str	2042.8	2151.0	2098.6	2245.9	2163.5
ν_14_ *a*′ CH str	3347.8	3355.9	3347.6	3496.5	3359.2
ν_15_ *a*′ OH str	3753.7	3754.9	3708.6	4001.5	3485.0
B state					
E(B) – E(X)		1.41	1.32(1.30^b^)	1.26	1.12
R(C≡C)		1.218	1.249	1.218	1.244
R(C–C)		1.453	1.503	1.432	1.512
R(C–O)		1.310	1.331	1.341	1.322
R(C=O)		1.225	1.205	1.241	1.201
ν_1_ *a*′ in-plane CCC bend		171.6	165.6	205.6	170.1
ν_2_ *a*″ out-of-plane CCC bend		201.1	197.7	263.6	201.5
ν_3_ *a*″ OH torsion		596.4	357.5	536.4	383.6
ν_4_ *a*′ CC–O bend		500.5	499.3	543.9	493.0
ν_5_ *a*′ CC=O bend		589.4	572.6	628.0	570.0
ν_6_ *a*″ out-of-plane CH bend		788.1	698.2	734.6	695.3
ν_7_ *a*′ in-plane CH bend		798.3	717.4	837.8	718.9
ν_8_ *a*″ CCO2 out-of-plane		747.5	742.9	881.0	728.4
ν_9_ *a*′ C–C str		887.2	836.3	899.3	824.8
ν_10_ *a*′ C–C str – OH bend		1250.3	1132.3	1144.8	1083.6
ν_11_ *a*′ C–C str + OH bend		1397.7	1413.9	1429.4	1354.4
ν_12_ *a*′ C≡C str + C=O str		2637.9	2006.8	2017.0	2008.4
ν_13_ *a*′ C≡C str – C=O str		2130.8	2232.5	2421.0	2274.9
ν_14_ *a*′ CH str		3450.4	3352.2	3532.3	3318.4
ν_15_ *a*′ OH str		3721.1	3696.8	3977.5	3556.6

a Qualitative descriptions of vibrational modes are based on the EOMCC-IP calculations.

b E­(A) – E­(X) calculated using EOMCC-IP/cc-pVQZ//EOMCC-IP/cc-pVDZ.

The cation conformers with ∠OCOH = 0° are more stable than with ∠OCOH = 180°, in agreement with experimental and computational studies on the neutral molecule.^51^ The NIST Chemistry WebBook^48^ value for IP of the X state (10.45 eV) has been superseded by a new experimental adiabatic IP (10.78 eV).^61,62^ This value is in very good agreement with CBS-QB3, G3, CBS-APNO calculations^61^ as well as the present CCSD, CCSD­(T), EOMCC-IP calculations. Some vibrational progressions have been observed in the photoelectron spectra^33,34^ but not enough for a full vibrational assignment. Figure 6 compares the CCSD, EOM-CCSD, CASSCF, and L-PDFT calculated frequencies with those calculated by EOMCC-IP. The frequencies calculated by CCSD, EOMCCSD, EOMCC-IP, and CASSCF are in fairly good agreement with each other. The CASSCF frequencies tend to be a bit higher than the CC methods and L-PDFT shifts the frequencies closer to the CC values. Since the X, A, and B states are formed by removing an electron from the CC triple bond and the carbonyl bond, modes involving C≡C and C=O stretching (ν_12_ and ν_13_) have the largest variation. For the B state, the four computational methods obtain 2131, 2233, 2421, and 2275 cm^–1^ for the C≡C str – C=O str vibration. However, for the C≡C str + C=O str vibration, EOMCC yields 2638 cm^–1^ compared to 2007, 2017, and 2008 cm^–1^ with EOMCC-IP, CASSCF, and L-PDFT, respectively. This suggests that the 2638 cm^–1^ value is most likely an artifact of the EOMCC calculation, and that multireference methods may be needed for the B state of propiolic acid cation.

**Figure 6:**
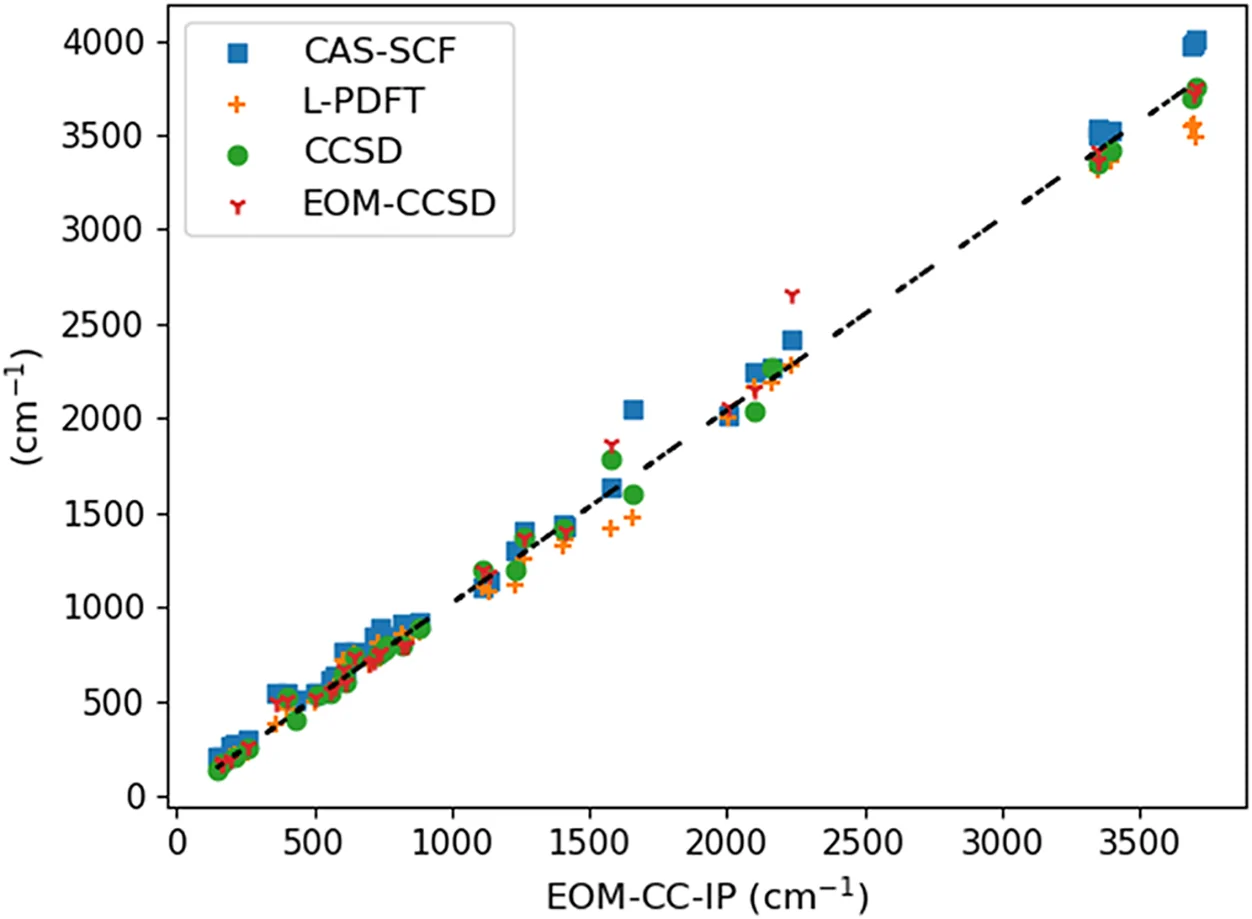
Harmonic vibrational frequencies (cm^–1^) of the propiolic acid cation calculated with CCSD, EOM-CCSD, CASSCF, and L-PDFT compared with calculated EOMCC-IP frequencies.

## Summary

A set of molecules of interest in charge migration studies has been selected to illustrate some of the computational challenges in calculating excited-state vibrational frequencies for cations. Haloacetylene cations are linear and should have degenerate bending vibrational frequencies. In spin-unrestricted CCSD and EOM-CCSD calculations, these frequencies are split because bending modes parallel to the unpaired electron see a different potential than that of perpendicular modes. Because the A states of the haloacetylene cations have the same symmetry as the X states, they need to be calculated by EOM-CCSD rather than CCSD. EOMCC-IP calculations employ the orbitals of the neutral molecule and, hence, are able to reproduce the degeneracy of the bending vibrations and are suitable for both the X and A states. State-averaged CASSCF also yields degenerate bending modes. MC-PDFT calculations using state-averaged CASSCF wave function results in large splittings in the bending frequencies; L-PDFT reduces the splitting by more than an order of magnitude and yields acceptable results.

Methyl halide cations also have degenerate ground states. Jahn–Teller interactions occur between the degenerate electronic states and the degenerate vibrational modes. For single-reference methods (e.g., HF, DFT, CCSD, EOMCC-IP), the *C*_3*v*_ symmetry molecular structure distorts to *C*_*s*_ symmetry, resulting in large frequency splittings between the degenerate rocking modes and between the degenerate deformation modes. State-averaged CASSCF calculations restore the expected degeneracies in the vibrational modes. However, CASSCF calculations require careful choice of the active space, and achieving reliable results can be challenging even for small molecules. Adding electron correlation by L-PDFT improves the agreement with experimental frequencies.

For vinyl bromide cation and propiolic acid cation, charge migration involves the coherent superposition of the ground state and the second excited state. Since the second excited state of these cations have the same symmetry as the ground state, they cannot be calculated by CCSD. EOM-CCSD calculations of the structure and vibrational frequencies are suitable but are costly because full CC calculations (*O*(*N* ^6^) work) are needed for every step in the numerical differentiation of the gradients needed for the computation of the second derivatives and vibrational frequencies. EOMCC-IP calculations are less expensive, with the energy of all of the states requiring only *O*(*N* ^5^) work after one initial CCSD calculation of the neutral ground state. The present work used numerical differentiation of the energy to obtain the first and second derivatives needed for geometry optimization and vibrational frequencies. Analytic gradients for EOMCC-IP have been formulated and incorporated in a few codes. Wider availability of analytic gradients will make EOMCC-IP more attractive for calculating structures and potential energy surfaces for cations since it is a simple-to-use “black box” approach.

The calculated harmonic vibrational frequencies are typically higher than the observed frequencies because of vibrational anharmonicity. The basis set size and method of including for electron correlation also contribute to the difference between the calculated and observed frequencies. As described above, these systematic differences can be taken into account by multiplying the calculated frequencies by a uniform scaling factor, obtained by a least-squares fit between calculated and experimental frequencies. Table 6 summarizes the scaling factors and the mean absolute percent deviation (MAPD) of the scaled calculated frequencies compared to the experimental frequencies. These provide a measure of the quality of the calculated frequencies. The experimental vibrational frequencies of the neutral molecules are reproduced quite well by the scaled CCSD/cc-pVTZ calculated frequencies, which have a MAPD of less than 1.5%. The MAPDs for neutral molecules calculated with CASSCF and MC-PDFT are somewhat higher, 1.8–4.2%. For the cations, the MAPDs are noticeably higher, 3.5–7.7%, reflecting the difficulty of calculating the electronic structure of the excited states of the cations. Unrestricted CCSD and EOM-CCSD are prone to splitting of vibrational frequencies that should be degenerate. EOMCC-IP has the advantage of treating the ground state and excited states of the cations on an equal footing and is a good choice for single-reference systems. However, EOMCC-IP can still have problems with Jahn–Teller cases. State-averaged CASSCF is suitable for multireference and Jahn–Teller cases. Adding electron correlation with MC-PDFT and L-PDFT reduces the MAPDs and improves the calculated frequencies compared to CASSCF.

**Table 6:** Vibrational Frequency Scale Factors and Mean Absolute Percent Deviation of Scaled Frequencies Compared to Experiment for Haloacetylenes, Methyl Halides, Vinyl Bromide, and Propiolic Acid Neutrals and Cations

	CCSD EOM-CCSD	EOMCC-IP	CASSCF	MC-PDFT L-PDFT
HCCX and CH_3_X	0.9547		0.9861	0.9857
	1.4%		1.8%	4.2%
H_2_C=CHBr and HC≡CCO_2_H	0.9531		0.9430	0.9828
	0.9%		3.5%	2.9%
HCCX^+^	0.9316	0.9367	0.9860	0.9683
	7.2%	6.4%	7.7%	4.8%
CH_3_X^+^	0.9663	0.9672	0.9747	0.9918
	5.5%	5.4%	4.7%	6.1%
H_2_C=CHBr^+^	0.9421	0.9474	0.9057	0.9740
	3.8%	3.5%	8.7%	5.5%

## Supplementary Material



## Notes

Vibrational frequencies of HCCI and CH_3_I neutral and cations with HF, B3LYP, MP2, CCSD, and EOMCCSD-IP and the cc-pVDZ, cc-pVTZ, and cc-pVQZ basis sets; Cartesian coordinates for cations of HCCI, HCCBr, CH_3_I, CH_3_Br, vinyl bromide, and propiolic acid optimized with EOMCCSD-IP (PDF)
