# Supplementary material for: Charge Migration in Cations: Structures, Energies, and Vibrational Frequencies of Ground and Excited States of HCCX+, CH3X+, Vinyl Bromide Cation and Propiolic Acid Cations
Source: J Phys Chem A. 2026 Sep 10;130(38):7544–58. doi: 10.1021/acs.jpca.6c02409 (PMC13618038; doi:10.1021/acs.jpca.6c02409)
Supplement: Supplementary file 1 [file jp6c02409_si_001.pdf]

Charge migration in cations: structures, energies and vibrational frequencies of ground and excited states of $HCCX^+$, CH_3X^+, vinyl bromide cation and propiolic acid cations

Supporting Information

Deep Mukherjee,[1] Andrew S. Durden,[1] Matthew R. Hermes,[2] Marco Caricato[3]

and H. Bernhard Schlegel[1 *]

[1] Department of Chemistry, Wayne State University, Detroit, MI 48202

[2] Department of Chemistry, University of Chicago, Chicago, IL 60637

[3] Department of Chemistry, University of Kansas, Lawrence, KS 66045

Table S1. Method and Basis Set Dependence for HCCI

HF	cc-pVDZ	cc-pVTZ	cc-pVQZ
R(CI)	2.007042	1.998200	1.995557
R(CC)	1.192974	1.181900	1.180836
R(CH)	1.063996	1.054200	1.054310
ν_1 π	307.6377	319.9311	318.7392
ν_2 σ	540.9445	548.7788	548.2453
ν_3 π	804.0830	825.5992	821.9757
ν_4 σ	2339.5476	2323.1458	2324.2417
ν_5 σ	3634.2759	3616.2592	3608.7840

B3LYP	cc-pVDZ	cc-pVTZ	cc-pVQZ
R(CI)	2.008320	1.999486	1.996470
R(CC)	1.213969	1.201036	1.200112
R(CH)	1.072496	1.061566	1.061407
ν_1 π	259.1160	280.6507	280.1057
ν_2 σ	511.4669	517.2547	517.4382
ν_3 π	648.4898	673.9939	667.8471
ν_4 σ	2158.0318	2148.8216	2148.2004
ν_5 σ	3476.3999	3467.9132	3460.6576

MP2	cc-pVDZ	cc-pVTZ	cc-pVQZ
R(CI)	2.002237	1.981650	1.967450
R(CC)	1.233687	1.217831	1.214134
R(CH)	1.075921	1.061726	1.061575
ν_1 π	251.4661	282.5739	290.6336
ν_2 σ	522.4762	539.5492	544.7265
ν_3 π	610.2275	632.5992	641.4486
ν_4 σ	2056.0308	2051.5866	2063.0533
ν_5 σ	3493.9333	3482.6809	3483.4829

CCSD	cc-pVDZ	cc-pVTZ	cc-pVQZ
R(CI)	2.014012	1.993300	1.981417
R(CC)	1.224412	1.206118	1.202282
R(CH)	1.077302	1.061680	1.061680
ν_1 π	259.8709	287.3327	293.8439
ν_2 σ	513.4734	531.6955	535.5746
ν_3 π	639.4042	674.4502	685.4456
ν_4 σ	2138.0217	2150.2693	2162.2315
ν_5 σ	3478.9530	3480.2410	3481.1271

Table S2. Method and Basis Set Dependence for $HCCl^+$ X State

HF	cc-pVDZ	cc-pVTZ	cc-pVQZ
R(Cl)	1.924685	1.917553	1.914197
R(CC)	1.226203	1.212353	1.210535
R(CH)	1.072909	1.062561	1.062458
ν_1	276.6452	284.7106	281.4532
ν_2	284.8688	297.9263	295.9827
ν_3 σ	602.7835	619.7511	620.7045
ν_4	755.8617	794.5672	797.8482
ν_5	764.0297	796.3456	798.1243
ν_6 σ	2002.9623	1996.8942	1996.3030
ν_7 σ	3528.3918	3516.6587	3511.7719

B3LYP	cc-pVDZ	cc-pVTZ	cc-pVQZ
R(Cl)	1.949594	1.942327	1.938521
R(CC)	1.238424	1.224164	1.223072
R(CH)	1.082751	1.071034	1.070676
ν_1	252.2239	260.8263	260.1330
ν_2	257.9023	274.0669	274.0540
ν_3 σ	558.2018	564.5292	564.4203
ν_4	654.5269	671.1589	672.1859
ν_5	755.9581	765.7791	767.4396
ν_6 σ	2004.7808	2004.3985	2001.9244
ν_7 σ	3380.5333	3377.6237	3372.4920

MP2	cc-pVDZ	cc-pVTZ	cc-pVQZ
R(Cl)	1.943565	1.928971	1.913462
R(CC)	1.226183	1.213253	1.210686
R(CH)	1.084919	1.071205	1.071041
ν_1	270.0091	289.6336	305.1128
ν_2	344.6225	353.4519	368.5621
ν_3 σ	616.2164	579.8250	592.4158
ν_4	804.1056	799.7254	810.4436
ν_5	925.8365	936.1646	948.1708
ν_6 σ	2396.4266	2358.2304	2375.1525
ν_7 σ	3474.1522	3459.4665	3452.4466

CCSD	cc-pVDZ	cc-pVTZ	cc-pVQZ
R(Cl)	1.945515	1.928718	1.914948
R(CC)	1.246225	1.227461	1.223748
R(CH)	1.086427	1.070741	1.070429
ν_1	254.0572	258.8734	274.7055
ν_2	255.8023	271.2507	286.7786

ν_3 σ	567.1460	584.4680	594.4439
ν_4	650.3536	676.5094	691.5912
ν_5	746.6021	762.4484	781.2451
ν_6 σ	1999.3099	2014.6335	2024.3289
ν_7 σ	3397.2502	3404.3061	3398.5865

EOMCCSD-IP	cc-pVDZ	cc-pVTZ	cc-pVQZ
R(CI)	1.9503	1.9342	1.919424
R(CC)	1.2402	1.2223	1.219006
R(CH)	1.0865	1.0707	1.070473
ν_1 π	245.4531	264.7620	285.5407
ν_2 π	245.4588	264.7665	286.3969
ν_3 σ	566.3943	582.2899	591.6054
ν_4 π	655.3197	684.2288	704.5029
ν_5 π	655.3298	684.2298	706.0841
ν_6 σ	2034.9171	2046.7434	2055.6876
ν_7 σ	3396.6871	3406.2985	3401.1474

Table S3. Method and Basis Set Dependence for $HCCI^+$ A State

EOMCCSD-IP	cc-pVDZ	cc-pVTZ	cc-pVQZ
R(CI)	2.1220	2.0957	2.081737
R(CC)	1.2507	1.2327	1.228267
R(CH)	1.0883	1.0723	1.071793
ν_1 π	221.7541	249.4286	268.4603
ν_2 π	221.7569	249.4427	268.5244
ν_3 σ	425.5227	448.9175	454.1872
ν_4 π	644.6012	678.3129	702.6109
ν_5 π	644.6065	678.3498	702.7146
ν_6 σ	2086.0123	2104.6087	2116.5098
ν_7 σ	3378.8589	3389.2883	3387.1569

Table S4. Method and Basis Set Dependence for CH_3I (C_{3v})

HF	cc-pVDZ	cc-pVTZ	cc-pVQZ
R(CI)	2.158806	2.144535	2.141748
R(CH)	1.084651	1.075124	1.074631
A(CH)	107.372	107.522	107.562
ν_1 A1	549.6316	566.4675	565.2126
ν_2 E	962.3802	975.3736	975.4013
ν_3 A1	1377.2151	1401.7270	1402.7286
ν_4 E	1564.4218	1588.2974	1588.9398
ν_5 A1	3240.6429	3224.1518	3224.3184
ν_6 E	3371.5950	3335.3246	3334.5031

B3LYP	cc-pVDZ	cc-pVTZ	cc-pVQZ
R(CI)	2.176266	2.164550	2.161321
R(CH)	1.095387	1.082871	1.082190
A(CH)	107.312	107.345	107.364
ν_1 A1	515.3977	525.3431	524.9261
ν_2 E	877.2856	891.4351	892.2526
ν_3 A1	1249.3248	1276.3241	1277.2445
ν_4 E	1437.3787	1470.3869	1470.2094
ν_5 A1	3081.8718	3086.6341	3085.0305
ν_6 E	3207.0290	3195.3734	3193.7892

MP2	cc-pVDZ	cc-pVTZ	cc-pVQZ
R(CI)	2.150240	2.123684	2.114054
R(CH)	1.097743	1.082698	1.081528
A(CH)	107.937	107.998	107.891
ν_1 A1	555.8708	581.3640	577.5754

ν_2 E	903.1292	910.9597	909.8345
ν_3 A1	1290.5758	1302.1439	1291.9227
ν_4 E	1470.2883	1485.2586	1484.8359
ν_5 A1	3123.8094	3119.9631	3123.0622
ν_6 E	3255.9214	3239.9369	3244.6717

CCSD	cc-pVDZ	cc-pVTZ	cc-pVQZ
R(CI)	2.161910	2.134477	2.123019
R(CH)	1.099124	1.083172	1.082071
A(CH)	107.845	107.939	107.906
ν_1 A1	537.5326	565.4064	566.5859
ν_2 E	897.9993	909.7587	911.2914
ν_3 A1	1290.7622	1309.4244	1304.9542
ν_4 E	1470.5528	1489.7429	1491.7228
ν_5 A1	3108.2876	3109.3305	3113.5683
ν_6 E	3230.0783	3219.5429	3224.7413

Table S5. Method and Basis Set Dependence for CH_3I^+ X State (^{2}A' C_s)

HF	cc-pVDZ	cc-pVTZ	cc-pVQZ
R(CI)	2.222379	2.189538	2.186129
R(CH1)	1.087051	1.078435	1.078008
R(CH2)	1.084037	1.074508	1.074056
A(CH1)	102.681	103.194	103.255
A(CH2)	104.497	105.184	105.275
D(CH2)	119.128	119.042	119.040
ν_1 A'	393.1379	429.2705	429.9223
ν_2 A”	453.6282	480.0549	482.1109
ν_3 A'	929.5388	941.3759	941.4357
ν_4 A'	1366.7208	1395.9450	1397.0764
ν_5 A”	1386.4077	1431.3763	1437.5489
ν_6 A'	1530.3585	1554.7102	1556.5538
ν_7 A'	3231.3682	3213.0319	3212.8014
ν_8 A”	3346.2291	3300.8698	3299.5268
ν_9 A'	3388.1166	3343.2748	3341.3573

B3LYP	cc-pVDZ	cc-pVTZ	cc-pVQZ
R(CI)	2.176844	2.160088	2.156387
R(CH1)	1.104323	1.092307	1.091670
R(CH2)	1.096858	1.084377	1.083724
A(CH1)	103.071	103.301	103.345
A(CH2)	106.198	106.444	106.525
D(CH2)	118.127	118.177	118.172
ν_1 A”	422.6035	435.6309	434.4338

ν_2 A’	439.9479	459.1243	459.5416
ν_3 A'	815.9635	830.5399	830.6007
ν_4 A”	1195.0527	1231.8117	1234.9716
ν_5 A’	1240.0910	1271.5410	1272.4491
ν_6 A'	1389.4067	1422.3259	1423.3337
ν_7 A'	3027.4873	3031.9786	3030.0758
ν_8 A”	3138.3166	3121.8757	3119.4718
ν_9 A'	3160.3810	3149.7181	3147.3999

MP2	cc-pVDZ	cc-pVTZ	cc-pVQZ
R(CI)	2.150286	2.115260	2.104204
R(CH1)	1.103907	1.090525	1.089752
R(CH2)	1.098278	1.083660	1.082641
A(CH1)	103.735	103.788	103.665
A(CH2)	106.758	107.183	107.190
D(CH2)	118.433	118.265	118.234
ν_1 A”	488.3350	511.3562	514.7270
ν_2 A’	493.8840	531.5513	528.6451
ν_3 A'	856.4576	856.6332	854.7404
ν_4 A'	1291.1078	1304.6556	1298.2513
ν_5 A”	1304.5330	1325.0746	1324.7418
ν_6 A'	1438.5672	1451.2832	1450.5659
ν_7 A'	3090.3434	3079.0167	3078.7724
ν_8 A”	3216.2119	3192.5099	3193.8519
ν_9 A'	3230.5304	3204.2673	3205.1722

CCSD	cc-pVDZ	cc-pVTZ	cc-pVQZ
R(CI)	2.176333	2.136703	2.125513
R(CH1)	1.104225	1.089929	1.089166
R(CH2)	1.099173	1.083784	1.082817
A(CH1)	103.483	103.670	103.613
A(CH2)	106.183	106.686	106.731
D(CH2)	118.569	118.428	118.404
ν_1 A”	482.6175	489.3887	498.3799
ν_2 A’	459.3730	499.8249	509.5193
ν_3 A'	853.2683	860.5035	860.8011
ν_4 A'	1284.5207	1306.4153	1303.8321
ν_5 A”	1301.4853	1326.6495	1337.1254
ν_6 A'	1436.0835	1453.4066	1454.6318
ν_7 A'	3084.9397	3077.2557	3078.4298
ν_8 A”	3202.7012	3181.3242	3179.9231
ν_9 A'	3218.6740	3195.8293	3197.7504

EOMCCSD-IP	cc-pVDZ	cc-pVTZ	cc-pVQZ
R(CI)	2.1826	2.1389	2.12503
R(CH1)	1.1040	1.0899	1.08943
R(CH2)	1.0988	1.0833	1.08260
A(CH1)	103.161	103.410	103.502
A(CH2)	105.930	106.560	106.650
D(CH2)	118.562	118.360	118.322
ν_1 A”	465.5791	487.6418	459.9836
ν_2 A’	446.5630	492.9495	495.4764
ν_3 A'	854.3459	861.7891	857.6703
ν_4 A'	1281.5379	1302.8364	1298.5328
ν_5 A”	1283.7363	1303.6579	1300.6911
ν_6 A'	1433.2892	1449.8303	1449.3931
ν_7 A'	3085.5715	3077.1205	3075.6695
ν_8 A”	3205.6744	3183.9636	3178.9500
ν_9 A'	3225.0040	3201.3663	3199.6345

Table S6. Method and Basis Set Dependence for CH_3I^+ A State (C_{3v})

HF	cc-pVDZ	cc-pVTZ	cc-pVQZ
R(CI)	3.003657	2.793114	2.790259
R(CH)	1.087256	1.076159	1.075467
A(CH)	91.536	92.699	92.738
ν_1 A1	83.5902	131.8145	136.3160
ν_2 E	399.3476	499.1728	504.4299
ν_3 A1	1412.3288	1389.7699	1388.1260
ν_4 E	1502.2861	1524.8565	1527.1707
ν_5 A1	3217.5664	3206.8645	3209.7702
ν_6 E	3443.3017	3408.3487	3410.3376

B3LYP	cc-pVDZ	cc-pVTZ	cc-pVQZ
R(CI)	2.634514	2.593821	2.593592
R(CH)	1.098292	1.085139	1.084407
A(CH)	94.056	94.267	94.252
ν_1 A1	217.7507	237.2989	237.1073
ν_2 E	436.1404	461.3265	464.1596
ν_3 A1	1206.0502	1225.7485	1225.9781
ν_4 E	1377.4824	1409.4129	1410.8191
ν_5 A1	3046.1988	3057.4205	3057.6808
ν_6 E	3251.9658	3241.5098	3240.9570

MP2	cc-pVDZ	cc-pVTZ	cc-pVQZ
R(CI)	2.546566	2.467769	2.462268
R(CH)	1.098599	1.084137	1.083077

A(CH)	94.966	95.327	95.169
ν_1 A1	271.7365	314.1174	313.5933
ν_2 E	546.2812	564.9099	566.2329
ν_3 A1	1251.0178	1258.5187	1258.5407
ν_4 E	1423.6534	1435.3138	1434.3435
ν_5 A1	3104.2206	3095.6727	3097.0766
ν_6 E	3320.4667	3290.8339	3293.0062

CCSD	cc-pVDZ	cc-pVTZ	cc-pVQZ
R(CI)	2.594063	2.511381	2.505727
R(CH)	1.100977	1.085286	1.084204
A(CH)	94.519	94.965	94.832
ν_1 A1	245.2909	283.3334	283.5878
ν_2 E	524.7839	550.7377	554.0221
ν_3 A1	1237.1285	1249.5096	1250.9043
ν_4 E	1417.5132	1432.9147	1432.1524
ν_5 A1	3083.9364	3080.7084	3083.5735
ν_6 E	3290.1106	3268.2267	3272.1358

EOMCCSD-IP	cc-pVDZ	cc-pVTZ	cc-pVQZ
R(CI)	2.575714	2.497358	2.49104
R(CH)	1.100518	1.084816	1.08376
A(CH)	94.660	95.120	95.01481
ν_1 A1	246.1434	284.4179	284.0633
ν_2 E	548.8024	562.7507	572.6664
ν_3 A1	1257.5190	1265.7138	1265.3354
ν_4 E	1419.6655	1432.6518	1434.0277
ν_5 A1	3086.4442	3083.7561	3087.5178
ν_6 E	3297.3591	3275.0701	3280.1022

Cartesian coordinates (angstrom)

HCCI neutral ground state - CCSD/cc-pVTZ
H 0.00000000 0.00000000 -3.72627100
C 0.00000000 0.00000000 -2.66446900
C 0.00000000 0.00000000 -1.45848000
I 0.00000000 0.00000000 0.53705600

HCCI+ X state - EOMCCSD-IP/cc-pVTZ
I 0.00000000 0.00000000 0.52683900
C 0.00000000 0.00000000 -1.40736100
C 0.00000000 0.00000000 -2.62966100
H 0.00000000 0.00000000 -3.70036100

HCCI+ A state - EOMCCSD-IP/cc-pVTZ
I 0.00000000 0.00000000 0.55977700
C 0.00000000 0.00000000 -1.53592300
C 0.00000000 0.00000000 -2.76862300
H 0.00000000 0.00000000 -3.84092300

HCCBr neutral ground state - CCSD/cc-pVTZ
H 0.00000000 0.00000000 -3.37515500
C 0.00000000 0.00000000 -2.31396800
C 0.00000000 0.00000000 -1.11030100
Br 0.00000000 0.00000000 0.68345100

HCCBr+ X state - EOMCCSD-IP/cc-pVTZ
H 0.00000000 0.00000000 -3.35973700
C 0.00000000 0.00000000 -2.28686100
C 0.00000000 0.00000000 -1.05621900
Br 0.00000000 0.00000000 0.66909200

HCCBr+ A state - EOMCCSD-IP/cc-pVTZ
H 0.00000000 0.00000000 -3.47742100
C 0.00000000 0.00000000 -2.40608900
C 0.00000000 0.00000000 -1.18710300
Br 0.00000000 0.00000000 0.71533100

CH3I neutral ground state - CCSD/cc-pVTZ
C 0.00000000 0.00000000 -1.80900600
I 0.00000000 0.00000000 0.32602400
H 0.00000000 1.03070600 -2.14173700
H 0.89261800 -0.51535300 -2.14173700
H -0.89261800 -0.51535300 -2.14173700

CH3I+ X state - EOMCCSD-IP/cc-pVTZ
C 1.81517400 0.00000200 0.00603700
I -0.32464900 0.00000000 0.00034300
H 2.06991700 -0.00016600 -1.05370600
H 2.12271200 -0.91393100 0.49979200
H 2.12270800 0.91408500 0.49951600

CH3I+ A state - EOMCCSD-IP/cc-pVTZ
C 2.12917300 0.00000500 0.00000600
I -0.36703700 0.00000000 0.00000000
H 2.22597400 -0.73599100 0.79106500
H 2.22600000 1.05309400 0.24179700

H 2.22595000 -0.31714500 -1.03290600

CH3Br neutral ground state - CCSD/cc-pVTZ
C 1.51646600 0.00007200 0.00003300
Br -0.41877700 -0.00000400 -0.00000200
H 1.85267400 1.01353700 0.18294800
H 1.85290800 -0.34839800 -0.96913100
H 1.85281900 -0.66542700 0.78606300

CH3Br+ X state - EOMCCSD-IP/cc-pVTZ
C -1.52390800 0.00124800 0.00906500
Br 0.41648400 0.00006800 0.00075900
H -1.83366000 -0.89343800 0.53791800
H -1.83712100 0.94513400 0.43950000
H -1.76270800 -0.06154900 -1.05836900

CH3Br+ A state - EOMCCSD-IP/cc-pVTZ
C 0.00000000 0.00000000 -1.89790900
Br 0.00000000 0.00000000 0.49404100
H 0.00000000 1.08309400 -1.96800000
H 0.93798700 -0.54154700 -1.96800000
H -0.93798700 -0.54154700 -1.96800000

C2H3Br neutral ground state - CCSD/cc-pVTZ
Br 0.00000000 0.72421300 0.00000000
C 0.45834700 -1.10413600 0.00000000
C -0.44269400 -2.07691800 0.00000000
H 1.52294400 -1.27552600 0.00000000
H -1.50422000 -1.87920100 0.00000000
H -0.11264500 -3.10641800 0.00000000

C2H3Br+ X state - EOMCCSD-IP/cc-pVTZ
Br 0.00000000 0.69933500 0.00000000
C 0.48802000 -1.03711400 0.00000000
C -0.46640300 -2.02658500 0.00000000
H 1.55074700 -1.23670800 0.00000000
H -1.52312300 -1.79343200 0.00000000
H -0.15732500 -3.06437700 0.00000000

C2H3Br+ A state - EOMCCSD-IP/cc-pVTZ
Br 0.72494500 -0.04646300 0.00000000
C -1.11556200 0.55865500 0.00000300
C -2.08516200 -0.33441800 -0.00000100
H -1.12893000 1.64000300 -0.00001100

H -1.94100300 -1.40460300 -0.00000400
H -3.09880800 0.04537200 0.00000200

C2H3Br+ B state - EOMCCSD-IP/cc-pVTZ
Br 0.76555200 -0.04544400 0.00495200
C -1.20267900 0.56124900 -0.00318900
C -2.18694100 -0.33613200 -0.00420500
H -1.22222300 1.63056700 -0.18636600
H -2.04996200 -1.39158200 -0.22548100
H -3.18442000 0.00085700 0.28290300

HC≡CCO2H neutral ground state - CCSD/cc-pVTZ
C -0.47197200 -2.11996400 0.00000000
C -0.23772400 -0.93997000 0.00000000
C 0.00000000 0.49597800 0.00000000
O 1.31222800 0.77663600 0.00000000
O -0.86722600 1.32406300 0.00000000
H 1.37867300 1.73945300 0.00000000
H -0.68051000 -3.16131300 0.00000000

HC≡CCO2H+ X state - EOMCCSD-IP/cc-pVDZ
C -0.02575600 -2.16078100 0.00000000
C -0.13970000 -0.93239400 0.00000000
C 0.00000000 0.48943900 0.00000000
O -1.00092800 1.29123300 0.00000000
O 1.21499900 0.78844500 0.00000000
H -0.72443000 2.23284600 0.00000000
H 0.00459900 -3.24785700 0.00000000

HC≡CCO2H+ A state - EOMCCSD-IP/cc-pVDZ
C -0.23579500 -2.18531600 0.00000000
C -0.04239000 -0.94689700 0.00000000
C 0.00000000 0.51647900 0.00000000
O 1.20087400 1.02618900 0.00000000
O -1.08668900 1.09353300 0.00000000
H 1.14034900 2.00378800 0.00000000
H -0.38472500 -3.26716300 0.00000000

HC≡CCO2H+ B state - EOMCCSD-IP/cc-pVDZ
C -0.53263600 -2.14132500 0.00000000
C -0.35089200 -0.90594200 0.00000000
C 0.00000000 0.55506100 0.00000000
O 1.33080900 0.59337200 0.00000000
O -0.76769500 1.48364100 0.00000000

H	1.61262100	1.53245800	0.00000000
H	-0.81635800	-3.19532600	0.00000000
